# Visualizing the chronicle of multiple cell fates using a near-IR dual-RNA/DNA–targeting probe

**DOI:** 10.1126/sciadv.adz6633

**Published:** 2025-10-22

**Authors:** Linawati Sutrisno, Gary J. Richards, Jack D. Evans, Michio Matsumoto, Xianglan Li, Koichiro Uto, Jonathan P. Hill, Masayasu Taki, Shigehiro Yamaguchi, Katsuhiko Ariga

**Affiliations:** ^1^ International Center for Young Scientists (ICYS), National Institute for Materials Science, 1-1 Namiki, Tsukuba, Ibaraki 305-0044, Japan.; ^2^ Research Center for Materials Nanoarchitectonics (MANA), National Institute for Materials Science, 1-1 Namiki, Tsukuba, Ibaraki 305-0044, Japan.; ^3^ School of Physics, Chemistry and Earth Sciences, The University of Adelaide, North Terrace, Adelaide, South Australia 5005, Australia.; ^4^ Bioanalysis Unit, National Institute for Materials Science, 1-2-1 Sengen, Tsukuba, Ibaraki 305-0047, Japan.; ^5^ Research Center for Macromolecules and Biomaterials, National Institute for Materials Science, 1-1 Namiki, Tsukuba, Ibaraki 305-0044, Japan.; ^6^ Institute of Transformative Bio-Molecules (WPI-ITbM), Nagoya University, Furo, Chikusa, Nagoya 464-8601, Japan.; ^7^ Institute for Glyco-core Research (iGCORE), Gifu University, 1-1 Yanagido, Gifu, Gifu 501-1193, Japan.; ^8^ Department of Advanced Material Science, Graduate School of Frontier Science, The University of Tokyo, 5-1-5 Kashiwanoha, Kashiwa, Chiba 277-8561, Japan.

## Abstract

Early detection and late-stage cell fate assessment are key factors to develop therapeutic strategies, although current methods cannot capture early responses or distinguish multiple injury states, especially in ultraviolet-visible (UV-vis)–sensitive cells. Here, we introduce a method to simultaneously detect variations in RNA and DNA under near-infrared photoexcitation. Using a pyrazinacene-based probe (**TEG**_**8**_**-N14**), we unexpectedly achieved discrimination of multiple cell states, including apoptosis, necrosis, necroptosis, and senescence, based on RNA/DNA changes. Specifically, **TEG**_**8**_**-N14** selectively stains necrotic cells in live samples, while after fixation, it allows detection of ultraearly senescence in UV-vis–sensitive cells, providing approximately twofold greater informational content than existing RNA or DNA fluorophores. These findings break current imaging barriers by enabling comprehensive visualization of single-cell fate histories without being affected by UV-vis or genetic manipulation.

## Teaser

Pyrazinacene’s unique structure enables various cell fate discrimination under dual NIR excitation.

## INTRODUCTION

Accurate monitoring of the fate of a single cell following the application of external stimuli is critical to the understanding of injury mechanisms and for the evaluation of therapeutic efficacy (*1*). However, current approaches for identifying cell fate transitions in heterogeneous cell populations fail simultaneously to detect different possible biological processes, often yielding inaccurate results, leading to an incomplete picture of cell fate posttreatment or erroneous conclusions in biological studies. For example, while caspase-3 is widely used as an apoptosis marker, it also participates in pathological processes, including oligodendrocyte injury in early multiple sclerosis lesions, confounding interpretation of imaging data (*2*, *3*). Similarly, acridine orange (AO), one of the few dyes capable of detecting early-stage apoptosis and necrosis, exhibits high cytotoxicity and requires ultraviolet-visible (UV-vis) excitation, rendering it incompatible with live or UV-vis–sensitive cells (*3*–*6*). Nuclear morphology is regarded as a reliable, universal senescence marker, although it provides limited sensitivity because of minimal morphological variations during early stages of senescence (*7*–*10*). An effective approach to assess single-cell fate transitions should meet the following criteria: (i) be operable in the near-infrared (NIR) excitation range (≥640 nm) for broad applicability even in UV-vis–sensitive cells, (ii) enable early detection of cell injury states, (iii) be highly informative in distinguishing different forms of cell injury posttreatment, (iv) support multiplex imaging for the analysis of complex biosystems, (v) exhibit superior sensitivity to cellular variations, and (vi) demonstrate exceptional photostability for prolonged imaging. Next-generation dyes should meet these criteria to enable accurate imaging results.

**Fig. 1.**
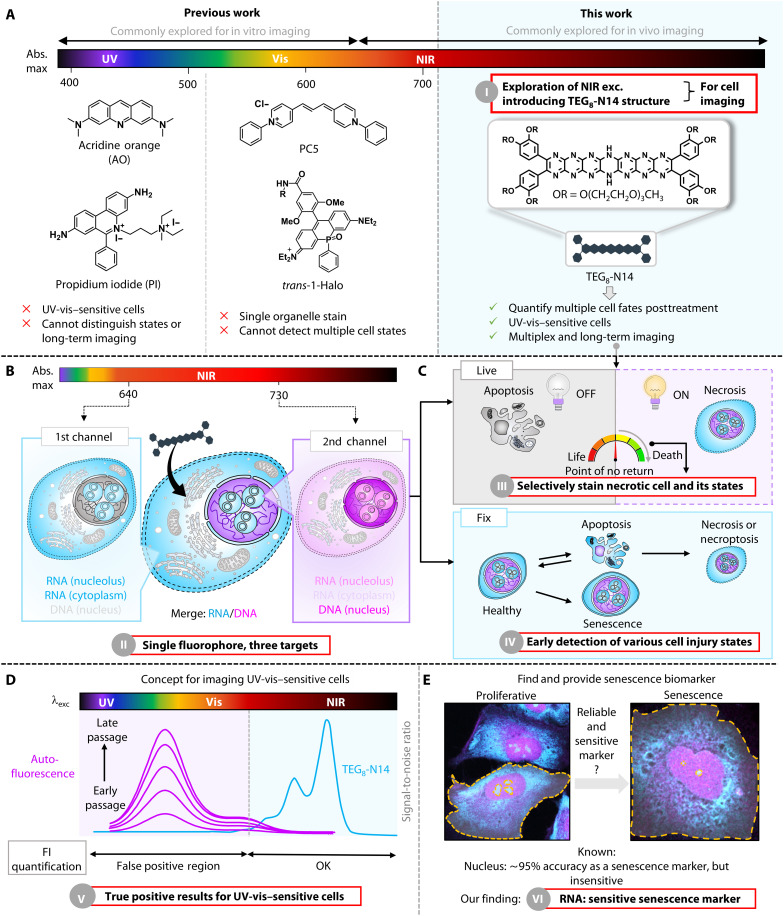
RNA-DNA and cell state discrimination using a single fluorophore under NIR excitation. (**A**) Comparison of **TEG**_**8**_**-N14** and conventional cellular dyes for detection of various cell injury states. (**B**) Illustration of simultaneous RNA-DNA visualization using **TEG**_**8**_**-N14** under dual NIR photoexcitation, enabling single-step staining for imaging complex biosystems and reducing the need for numerous control groups. (**C**) **TEG**_**8**_**-N14** exhibits different imaging behavior in live and fixed cellular systems. In live cells, **TEG**_**8**_**-N14** acts as a biomarker for irreversible necrosis, allowing for detailed, state-specific quantitative analysis of cell injury progression. In fixed cells, it facilitates precise discrimination among the states of apoptosis, necrosis, and necroptosis. (**D**) Our proposed concept to address the imaging barrier in UV-vis–sensitive cells. High autofluorescence and reactivity of endogenous fluorophores in ARPE-19 cells hinder the performance of existing UV-vis dyes, highlighting the need for **TEG**_**8**_**-N14** in FI quantification for this cell line. (**E**) Demonstration of RNA as a sensitive cell stress biomarker in ARPE-19 cells using **TEG**_**8**_**-N14**.

Here, we present a general strategy based on a class of *N*-heteroacene fluorophores, the pyrazinacenes, which have not so far been explored for use in bioimaging (*11*, *12*). We have explored the importance of NIR excitation of the pyrazinacene chromophore for cellular imaging and demonstrate the superiority of the probe over existing dyes (figs. S1 and S2 and Fig. 1A). Specifically, pyrazinacene (**TEG**_**8**_**-N14**) enables simultaneous RNA-DNA imaging under NIR excitation. Computational modeling revealed distinct interaction modes that contribute to the differential spectral responses of the probe in RNA-rich versus DNA-rich environments. The superior DNA-RNA sensitivity and exceptional photostability of **TEG**_**8**_**-N14** enable its application for precise detection of different states of cell injury (Fig. 1, B and C). To demonstrate this, we have used it to detect senescence in UV-vis–sensitive cells using autofluorescence-free channels (Fig. 1, D and E). Our findings reveal that compared to conventional nuclear morphology–based methods, RNA offers greater sensitivity for the prediction of cellular senescence. These findings establish a solid conceptual framework as a strategy to visualize the chronicle of single-cell fate transitions, surpassing the limitations of any currently available imaging systems.

## RESULTS

### Introducing pyrazinacene to the bioimaging field

To address the requirements for cell fate imaging, we used the tetradecaazaheptacene (N14) chromophore, which exhibits strong absorption in the biological transparency window (650 to 900 nm) (*13*). Given the nitrogen-rich, planar structure of the N14 core, we hypothesized that **TEG**_**8**_**-N14** might either operate as a DNA groove binder or interact with RNA chains through hydrogen bonding (as a donor or acceptor) or hydrophobic interactions (*13*, *14*). However, N14 chromophores are known to aggregate, causing aggregation-induced fluorescence emission quenching (*12*). As expected, **TEG**_**8**_**-N14** exhibits a peak absorbance in aqueous solution at around 664 nm and an extremely low quantum yield (below 0.01), caused by aggregation of the chromophores (fig. S3 and table S1). To modulate aggregation and enhance its emissive properties, **TEG**_**8**_**-N14** was added to an aqueous cetyltrimethylammonium bromide (CTAB) solution and used in subsequent comparison studies to investigate the effect of aggregation. In the presence of CTAB, pronounced enhancement of the 0-0 transition peak at ~730 nm was observed, accompanied by increases in molar absorption coefficient (_εPBS buffer_ =243,000 M^−1^ cm^−1^) and photoluminescence quantum yield (~0.23), indicating that interactions with CTAB induce fluorescence switch-on of **TEG**_**8**_**-N14** in aqueous solution because of changes in its state of aggregation. Spectral analysis reveals that these compounds are pH-sensitive (5.8 to 8.0) and remain stable at storage and application temperatures (figs. S4 and S5). Furthermore, we note that **TEG**_**8**_**-N14** in the presence of CTAB is one of the brightest NIR dyes for cellular imaging among the 13 popular NIR dyes reported to date (table S1) (*13*–*21*).

### RNA-DNA discrimination using a single fluorophore

**Fig. 2.**
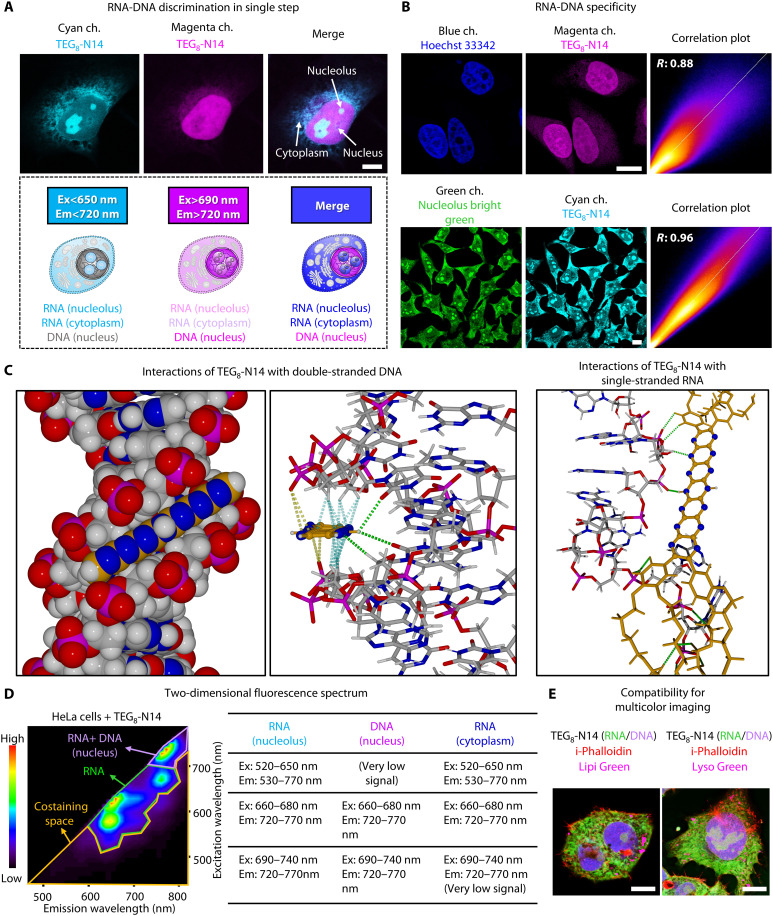
Performance of TEG_8_-N14 in fixed cell imaging. (**A**) Representative confocal images of a HeLa cell stained with **TEG**_**8**_**-N14** (upper) and proposed pseudocolor image in different channels (lower). See movies S1 to S4. Ex, excitation; Em, emission. Scale bar, 10 μm. (**B**) Representative costaining images (left and middle) and their colocalization analysis (right). DNA colocalization is observed in blue (λ_ex_ = 405 nm and λ_em_ = 415 to 600 nm) and magenta (λ_ex_ = 730 nm and λ_em_ = 740 to 850 nm) channels. RNA colocalization is observed in green (λ_ex_ = 450 nm and λ_em_ = 500 to 550 nm) and cyan (λ_ex_ = 640 nm and λ_em_ = 650 to 720 nm) channels. Scale bars, 10 μm. Single focal plane. *R* is the Pearson correlation coefficient. See fig. S8 for three-dimensional colocalization. (**C**) Docking simulation to reveal the interaction between **TEG**_**8**_**-N14** and dsDNA [left and middle; dotted lines indicate intermolecular interactions: green, hydrogen bonds (NH···O and CH···O); blue, CH···π; yellow, O···π (weak)] and ssRNA [right; green dotted lines indicate hydrogen bonding (CH···N, CH···O, and NH···O) interactions]. (**D**) Two-dimensional fluorescence imaging (left) and summary of excitation and emission wavelengths for detection of **TEG**_**8**_**-N14** for two targets [RNA (nucleolus and cytoplasm) and DNA (nucleus)]. (**E**) Four-color imaging of fixed HepG2 cells stained with **TEG**_**8**_**-N14** and several dyes (right). Red: i-Phalloidin (λ_ex_ = 405 nm and λ_em_ = 415 to 445 nm); magenta: Lipi Green (λ_ex_ = 470 nm and λ_em_ = 500 to 600 nm) or Lyso Green (λ_ex_ = 450 nm and λ_em_ = 500 to 600 nm); green: **TEG**_**8**_**-N14** (λ_ex_ = 640 nm and λ_em_ = 650 to 720 nm); purple: **TEG**_**8**_**-N14** (λ_ex_ = 730 nm and λ_em_ = 740 to 850 nm). Scale bars, 10 μm. All the cells were preserved in a fixed state, captured using volumetric mode, and conducted on at least two independent experiments.

Encouraged by the favorable optical properties of **TEG**_**8**_**-N14**, we evaluated its performance in cellular imaging. Cytotoxicity assays confirmed that **TEG**_**8**_**-N14** is essentially nontoxic below 10 μM, and this condition was selected for further live-cell imaging experiments (fig. S6). Unexpectedly, imaging of fixed HeLa cells treated with **TEG**_**8**_**-N14** revealed distinct staining patterns when excited at 640 or 730 nm (fig. S7 and Fig. 2A). On the basis of subcellular localization, we envisioned that signals excited at 640 nm originate predominantly from RNA-rich regions, while signals at 730 nm mainly correspond to DNA-rich regions. This observation indicates that **TEG**_**8**_**-N14** enables simultaneous staining of RNA and DNA in a single step. This dual labeling capability offers advantages for multiplexed imaging by reducing the number of control groups required. We further examined colocalization of **TEG**_**8**_**-N14** with RNA and DNA by costaining with a commercially available dye (Fig. 2B). Costaining with Hoechst 33342 revealed the high DNA specificity of **TEG**_**8**_**-N14** (*R* = 0.83 ± 0.06; *n* ≥ 21 cells), while RNA colocalization analysis yielded a Pearson correlation coefficient of 0.95 ± 0.01 (*n* ≥ 20 cells) with high overlap across all *z*-positions (fig. S8). The high specificity found here for RNA-DNA labeling is supported by the low degrees of overlap with different organelles (fig. S9A). Next, we measured the emission spectrum of the probe in HeLa cell lysate before and after nuclease treatment, followed by staining with **TEG**_**8**_**-N14** (fig. S9B). We found that in the spectra of total lysate with **TEG**_**8**_**-N14**, two absorbance peaks appeared at ~660 and 730 nm, consistent with the presence of a monomer and the aggregated state of the compound. In contrast, after deoxyribonuclease (DNase) and ribonuclease (RNase) treatment, we observed that the fluorescence intensity (FI) of the mixture was reduced. These results indicate that our probe selectively binds to cellular DNA or RNA rather than to other highly abundant biomolecules. On the basis of these results, we conclude that the probe is well suited for studying nucleic acids within cells with minimal interference involving nonspecific binding from other biomolecules. This finding is further supported by the fluorescence change of **TEG**_**8**_**-N14** obtained on the addition of commercially available double-stranded DNA (dsDNA), single-stranded DNA (ssDNA), and RNA, confirming their interaction with DNA and RNA within cellular systems (table S2 and figs. S10 and S11).

The molecular basis of the dual-binding behavior was investigated using docking simulations of **TEG**_**8**_**-N14** with representative dsDNA, single-stranded RNA (ssRNA), and double-stranded RNA (dsRNA) oligonucleotides. This revealed distinct interactions in a simulated implicit aqueous environment. The rigid planar structure of the N14 core preferentially inserts into the DNA minor groove (Fig. 2C, left and middle panels), where one NH group of the N14 unit interacts with carbonyl atoms of two thymine residues by hydrogen bonding. There are also multiple C─H···π interactions (*22*) involving methylene units of ribose in the DNA minor groove. A close approach of phosphate oxygen atoms also suggests anion-π interactions involving the anionic phosphate backbone and electron-deficient extremities of the N14 unit (*23*). In contrast, interactions with RNA are more variable and dependent on its secondary structure. ssRNA undergoes hydrogen bonding with the N14 core and TEG side chains, while dsRNA binding is mediated largely by nonspecific electrostatic interactions with the polyether appendages, with minimal π-π interaction (Fig. 2C, right, and fig. S12). These simulations explain the observed spectral behaviors. For DNA, groove binding encapsulates the molecule involving multiple intermolecular contacts, which has the effect of stabilizing the excited state relative to the ground state, leading to emission at a longer wavelength. More specifically, binding to DNA suppresses the geometry relaxation in the excited state because of the multiple interactions, leading to enhanced fluorescence emission at lower energy (i.e., enhanced 0-0 vibronic transition at ~730 nm). For RNA, intermolecular interactions depend more on disaggregation of **TEG**_**8**_**-N14**, leading to a higher energy of the excited state and emission at higher energy with an increased 0-1 transition intensity (fig. S13) (*24*, *25*).

Given its enhanced brightness, **TEG**_**8**_**-N14** in the presence of CTAB was selected for multicolor imaging and photostability assay (fig. S14). Two-dimensional fluorescence of **TEG**_**8**_**-N14** demonstrates its ability to minimize cross-talk by opening up an unpopular NIR excitation channel for multicolor imaging (Fig. 2, D and E, fig. S15, and movies S1 to S4). After confirming that staining with **TEG**_**8**_**-N14** with and without CTAB does not affect the staining pattern, we adopted a currently accepted standard method to compare the photostability of **TEG**_**8**_**-N14** with existing DNA or RNA probes based on half-lives of bleaching (figs. S16 and S17) (*14*). Photostability analysis shows that **TEG**_**8**_**-N14** has excellent photostability comparable to SiR-DNA, arguably the most photostable dye reported to date, highlighting its potential for volumetric imaging. We further explored the potential for application across various cell types. RNA-DNA fluorescence signals were observed at the same localizations in different cell types and morphologies, demonstrating a broad applicability across various cell lines (fig. S18). To the best of our knowledge, to date, this is the only reported probe that not only stains RNA and DNA simultaneously but also has high photostability under NIR photoexcitation (≥640 nm), making it suitable for use in studying diverse biological events.

### Broad applicability for measuring multiple single-cell injury states

Current microscopy techniques lack sensitivity to detect early apoptosis, rarely capture necrotic or necroptotic states, and typically require UV-vis excitation, limiting their applicability (*3*). Given the role of RNA as an early injury marker, we demonstrate here the capability of **TEG**_**8**_**-N14** to distinguish apoptosis, necrosis, and necroptosis. DNA and RNA sensitivity was confirmed through strong FI attenuation upon nuclease treatment (fig. S19), validating its responsiveness to DNA and RNA degradation. Spectral imaging of stained NIH-3T3 cells also confirms RNA and DNA detection in different channels, supporting RNA-DNA quantification for further study (fig. S17).

**Fig. 3.**
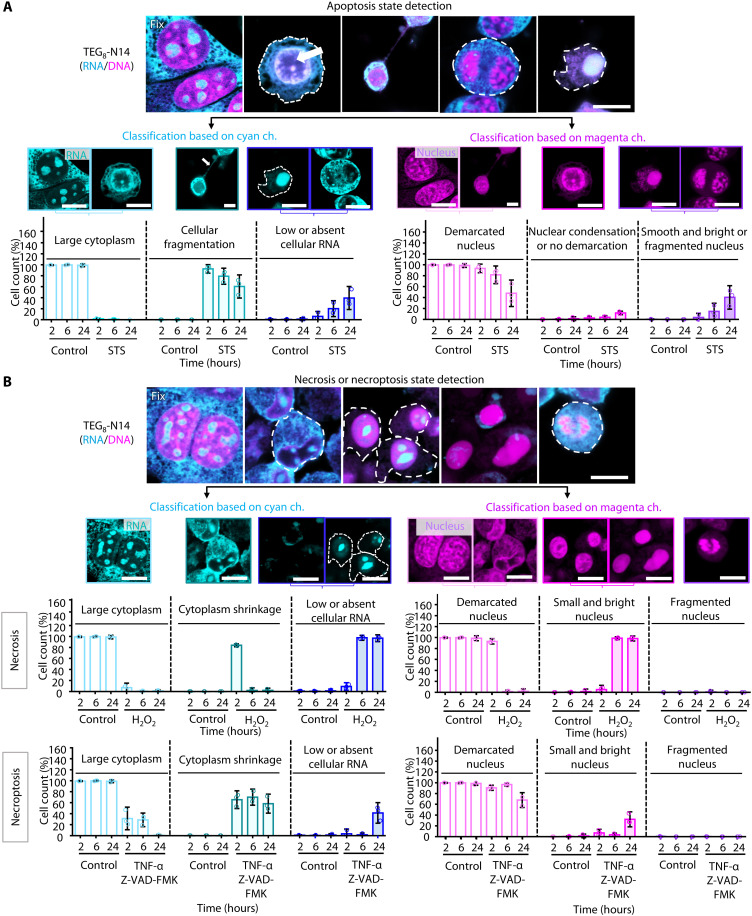
Single-cell analysis of different cell injury states after drug treatment for 2, 6, and 24 hours, followed by staining with TEG_8_-N14. (**A**) Representative confocal images of apoptotic states are shown (top), along with segmentation and analysis of apoptotic states based on cell morphology (bottom) in the cyan channel (λ_ex_ = 640 nm and λ_em_ = 650 to 720 nm) and magenta channel (λ_ex_ = 730 nm and λ_em_ = 740 to 850 nm). Scale bars, 10 μm. The white arrow indicates nuclear condensation; white dashes indicate RNA in the cytoplasm. Data presented are the means ± SD (*n* > 250 cells per experiment). The dotted indicates three independent experiments. Error bars represent SD. See fig. S21. STS, staurosporine. (**B**) Representative confocal images of necrosis and necroptosis states are shown (upper panels), and corresponding segmentation with the analysis of necrosis and necroptosis states based on the morphology of cells (lower panels) in the cyan channel (λ_ex_ = 640 nm and λ_em_ = 650 to 720 nm) and magenta channel (λ_ex_ = 730 nm and λ_em_ = 740 to 850 nm) is presented. Scale bars, 10 μm. White dashes indicate RNA in the cytoplasm. Data presented are the means ± SD (*n* > 250 cells per experiment). Dots indicate the three independent experiments. Error bars represent SD. All the cells were preserved in a fixed state, captured using volumetric mode, and conducted on at least two independent experiments.

To evaluate the use of **TEG**_**8**_**-N14** to distinguish different injury states, NIH-3T3 cells were treated with staurosporine to induce apoptosis, H_2_O_2_ to trigger necrosis, and TNF-α (tumor necrosis factor–α) combined with a caspase inhibitor to provoke necroptosis (*3*). In all cases, imaging after 6 hours revealed reduction in fluorescence intensities in both DNA and RNA channels (fig. S20). This result is consistent with the previous observation that nucleic acid degradation accompanies metabolic disruption during cell injury (*3*, *26*, *27*). Images were acquired at different times to identify distinct cell injury states using **TEG**_**8**_**-N14** at the single-cell level. Control cells exhibited flattened shapes with a cyan signal predominantly in the cytoplasm and a well-defined nucleus outlined fully by a magenta signal (fig. S21 and Fig. 3A). After 2 hours of staurosporine treatment, most cells lose their flattened shape, develop thin extensions, undergo cellular fragmentation, and show less distinct, shrunken nuclei. At advanced states of apoptosis, which might correspond to the onset of necrosis, the cyan signal in the cytoplasm is almost completely absent and nuclei are fragmented or show smooth and bright FI. In contrast to apoptosis, necrosis and necroptosis processes exhibit several distinct but mutually similar morphological features (Fig. 3B). At the early stages of these processes, there is a noticeable loss of cyan staining because of cellular shrinkage, although there is no cellular fragmentation. The initial signs include nuclear shrinkage and loss of distinct nuclear boundaries. At later stages of necrosis and necroptosis, nuclei shrink and often show increased intensity or a smooth appearance with minimal cytoplasmic cyan staining. The absence of cytoplasmic and nuclear fragmentation—features characteristic of apoptosis—along with the presence of a smooth, round nucleus with low levels of cytoplasmic RNA clearly differentiate necrosis and necroptosis from apoptosis. Notably, given that more than 80% of RNA is located in the cytoplasm, where it participates in protein synthesis and stress responses, it can act as a highly sensitive indicator of early cell injury (*3*). Thus, the responsivity of **TEG**_**8**_**-N14** to RNA allows detection of cell injury states that are challenging to resolve using conventional methods.

### Advantages of **TEG**_**8**_**-N14** as a necrotic state indicator over conventional methods

**Fig. 4.**
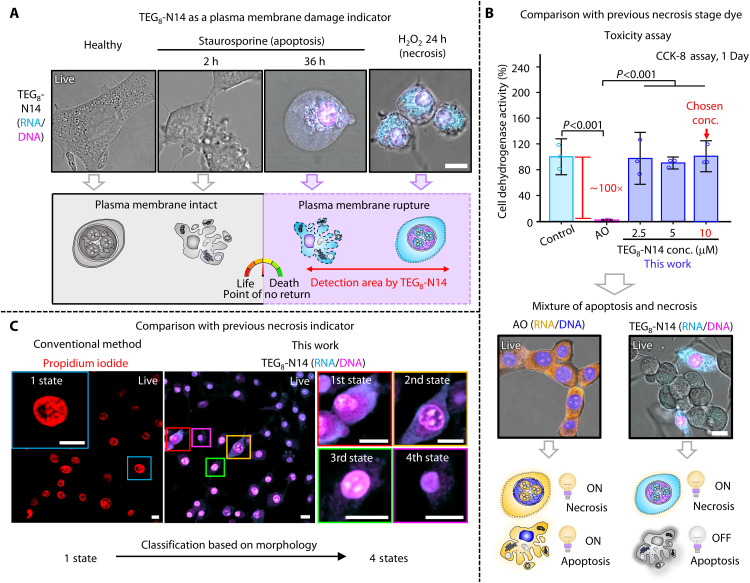
Comparison between TEG_8_-N14 and conventional necrosis dyes in live samples. (**A**) Representative confocal images of **TEG**_**8**_**-N14** as necrosis indicator in NIH-3T3 cells. Images were obtained in cyan (λ_ex_ = 640 nm and λ_em_ = 650 to 720 nm) and magenta (λ_ex_ = 730 nm and λ_em_ = 740 to 850 nm) channels. Scale bar, 10 μm. h, hours. (**B**) Comparison of existing commercial RNA-DNA dye (AO) and **TEG**_**8**_**-N14** for the detection of necrosis state in terms of cytotoxicity (upper) and necrosis detection (lower). Data presented are the means ± SD (*n* = 3). Error bars represent SD. Significance levels are determined using an unpaired two-tailed Student’s *t* test. Images were acquired in blue (λ_ex_ = 457 nm and λ_em_ = 467 to 550 nm), yellow (λ_ex_ = 457 nm and λ_em_ = 600 to 750 nm), cyan (λ_ex_ = 640 nm and λ_em_ = 650 to 720 nm), and magenta (λ_ex_ = 730 nm and λ_em_ = 740 to 850 nm) channels. Scale bar, 10 μm. (**C**) Comparison of existing necrosis indicator dye (PI) and **TEG**_**8**_**-N14** for the detection of necrosis state. Left image: The red color indicates PI (plasma membrane damage indicator). Right images: The cyan color indicates RNA stained with **TEG**_**8**_**-N14**, and the magenta color indicates a nucleus stained with **TEG**_**8**_**-N14**. Scale bars, 10 μm. All cells were directly stained without any fixative, imaged using volumetric mode, and conducted on at least two independent experiments.

To evaluate the ability of **TEG**_**8**_**-N14** to detect necrotic cells in live samples, we treated NIH-3T3 fibroblasts with staurosporine or hydrogen peroxide to induce necrosis or mixed apoptosis and necrosis (Fig. 4, A and B, and fig. S22). Fluorescence emission from **TEG**_**8**_**-N14** was observed only in cells with ruptured plasma membranes, thereby highlighting the potential of **TEG**_**8**_**-N14** as a selective indicator of necrotic states. Further validation with an efflux pump inhibitor in live samples confirmed that **TEG**_**8**_**-N14** cannot penetrate cells with intact plasma membranes (fig. S23), establishing that membrane rupture is required for probe entry. Traditionally, PI and AO have been gold standards for necrosis detection. However, both suffer from limitations: PI intercalates into DNA, exhibiting long-term toxicity, while AO requires UV-vis excitation, inducing phototoxicity (*28*, *29*), which restrict their use in live cells. In contrast to PI, which cannot be used to distinguish necrotic subtypes, **TEG**_**8**_**-N14** allows identification of necrotic states based on RNA sensitivity (Fig. 4C). Moreover, in comparison with AO, **TEG**_**8**_**-N14** exhibits ~100-fold lower cytotoxicity, allowing continuous imaging up to 24 hours coupled with the ability to detect necrotic cells (Fig. 4D and fig. S24). Overall, these results demonstrate that **TEG**_**8**_**-N14** outperforms conventional necrosis markers by providing selective detection of necrotic states, minimizing cytotoxicity, and enabling long-term and reliable tracking of cell death in live-cell imaging experiments.

### Finding a sensitive biomarker in senescence using an accurate detection method

**Fig. 5.**
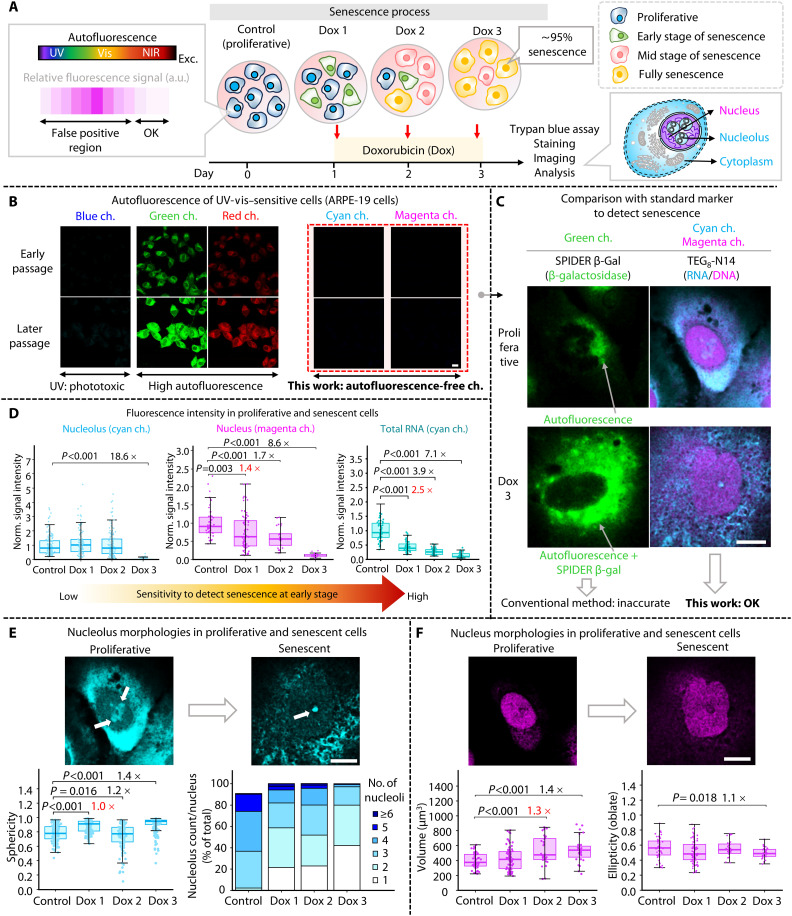
Reliable measurements in senescence-induced UV-vis–sensitive cells by TEG_8_-N14. (**A**) Schematic illustrations of chemically induced senescent cells among ARPE-19 cells and its imaging process. a.u., arbitrary units. (**B**) Representative confocal images of maximum intensity projection of endogenous fluorophores in ARPE-19 cells under different excitation conditions with an identical microscope setup. Blue channel: λ_ex_ = 405 nm and λ_em_ = 415 to 460 nm; green channel: λ_ex_ = 488 nm and λ_em_ = 500 to 550 nm; red channel: λ_ex_ = 561 nm and λ_em_ = 571 to 635 nm; cyan channel: λ_ex_ = 640 nm and λ_em_ = 650 to 720 nm; magenta channel: λ_ex_ = 730 nm and λ_em_ = 740 to 850 nm. Scale bar, 10 μm. Representative results for *n* = 3 over two independent experiments. (**C**) Representative confocal images of proliferative and senescent ARPE-19 cells stained with SPIDER β-Gal and **TEG**_**8**_**-N14**. Merged images were obtained in green (λ_ex_ = 488 nm and λ_em_ = 500 to 550 nm), cyan (λ_ex_ = 640 nm and λ_em_ = 650 to 720 nm), and magenta (λ_ex_ = 730 nm and λ_em_ = 740 to 850 nm) channels. (**D**) FI changes in the nucleolus (left), nucleus (middle), and total RNA (right) measured after treatment with Dox to induce senescence from day 1 to day 3. Data are presented as the means ± SD and plotted from three biological replicates. *n* > 30 cells by an unpaired two-tailed Student’s *t* test. Error bars represent SD. Three-dimensional morphological images (upper) and its analysis (lower) of the nucleolus (**E**) and nucleus (**F**). Data are presented as the means ± SD and plotted from three biological replicates. *n* > 30 cells. White arrows indicate the nucleolus. Error bars represent SD. Scale bar, 10 μm. Statistical significance was assessed using an unpaired two-tailed *t* test. All the cells were preserved in a fixed state, captured using volumetric mode, and conducted on at least two independent experiments.

Next, we explored the possibility of extending our classification strategy to detect cell senescence (*7*, *30*–*34*), a biological phenomenon whose detection is challenging. For this purpose, ARPE-19 cells were selected as a model because of their high level of endogenous fluorophores, which obstruct high reproducibility imaging (*5*, *35*–*37*). ARPE-19 cells were treated with doxorubicin (Dox), inducing ≥95% senescence (*38*–*40*), as verified by trypan blue assay (Fig. 5A). Before cell imaging, ARPE-19 cells were imaged using different excitation wavelengths to identify the optimum channel for observation. As expected, for excitation below 640 nm, ARPE-19 cells exhibited autofluorescence, which increased at higher passage numbers of the cells (Fig. 5B). In contrast, when cells were imaged under excitation above 640 nm even at the maximum laser power of the microscope, almost no autofluorescence was observed, confirming that NIR excitation channels are suitable for the accurate quantification of FI of this cell line.

Senescence in ARPE-19 cells is difficult to detect using conventional RNA/DNA dyes because of UV-vis excitation requirements, end-point limitations, and signal cross-talk. Although nuclear morphology is ~95% accurate, its low early-stage sensitivity highlights the need for better biomarkers (*3*, *7*, *41*). With the autofluorescence-free properties of **TEG**_**8**_**-N14** in mind, we simultaneously visualized RNA in the nucleolus, RNA in the cytoplasm, and the nuclear DNA. First, we compared our system with the conventional senescence marker SPIDER β-galactosidase (β-Gal) (Fig. 5C and fig. S25). As expected, SPIDER β-Gal imaging was subject to interference involving cross-talk between the dye and endogenous fluorophores in ARPE-19 cells. In contrast, **TEG**_**8**_**-N14** gave more accurate results for the easy detection of senescence because of the autofluorescence-free channel (Fig. 5, B and C). To the best of our knowledge, these results represent an initial report on RNA-DNA visualization of chemically induced senescence in ARPE-19 cells using dual autofluorescence-free channels.

On the basis of these results, we conducted an in-depth analysis using our system (fig. S26 and Fig. 5D). FI quantification showed a ~2.5-fold decrease in total RNA, surpassing changes in the nucleolus and nucleus at the earliest senescence state, indicating total RNA as a promising and sensitive senescence marker. Next, we performed quantitative analyses of morphological changes in the nucleus and nucleolus using volumetric segmentation analysis (Fig. 5, E and F, and figs. S27 and S28). We observed that the sphericity of the nucleolus increased, while the nucleolus counts per cell decreased, aligned with previous findings (*39*). In addition, the nucleus became larger and more flattened after the cells entered senescence, consistent with previous results (*7*). These findings imply that FI quantification of total RNA using **TEG**_**8**_**-N14** is the most sensitive method for detecting senescence in ARPE-19 cells, surpassing changes in the nucleolus and nucleus at the earliest states of senescence. **TEG**_**8**_**-N14** outperforms individual RNA or DNA dyes, given that it provides an approximately twofold higher information level, has superior RNA sensitivity, eliminates the requirement for UV-vis excitation, and can be used even in ARPE-19 cells. Our method also enables the distinction of senescence by combining a widely regarded reliable marker (nuclear morphology) with a sensitive marker (RNA) in a single step while providing accurate FI quantification.

## DISCUSSION

Precise determination of cell fate transitions in heterogeneous populations requires probes capable of sensitively and simultaneously monitoring multiple molecular states at the single-cell level (*3*, *42*–*45*). While both RNA and DNA play central roles in defining cellular states, conventional nucleic acid dyes are typically limited to a single target, rely on UV-vis excitation, and often suffer from phototoxicity and autofluorescence, particularly in sensitive or highly autofluorescent cell lines (*4*, *13*). Here, we demonstrate the usefulness of the pyrazinacene dye **TEG**_**8**_**-N14**, effectively overcoming the imaging barriers of conventional fluorophores. Our results highlight six key advantages of **TEG**_**8**_**-N14** over conventional RNA-DNA stains. These are as follows: (i) Its unique dual-staining capability enables the simultaneous visualization of RNA and DNA (Figs. 1 and 2B). This feature enhances usability by reducing the need for multiple control groups and provides a high informational level for the monitoring of biological events. (ii) By eliminating the need for UV-vis excitation, **TEG**_**8**_**-N14** avoids phototoxicity, autofluorescence, and spectral overlap, allowing its application in UV-vis–sensitive cells (Figs. 2D and 5B). (iii) It exhibits high photostability (fig. S16) for quantitative fluorescence imaging without photobleaching artifacts. (iv) Its RNA sensitivity enables the detection of a wide range of cell injury states, from the earliest to later states, including apoptosis, necrosis, and necroptosis (Fig. 3). (v) In live-cell imaging, **TEG**_**8**_**-N14** selectively accumulates in necrotic cells, providing a reliable platform for real-time necrosis tracking (Fig. 4). In contrast to PI and AO, which suffer from high cytotoxicity and low specificity, **TEG**_**8**_**-N14** allows for long-term imaging up to 24 hours. (vi) In ARPE-19 cells, **TEG**_**8**_**-N14** offers autofluorescence-free quantification of RNA and nuclear morphology, outperforming standard senescence markers for sensitivity and resolution (Fig. 5).

Overall, these advantages position **TEG**_**8**_**-N14** as a next-generation multifunctional probe. Beyond its technical performance, our findings demonstrate that RNA is a highly sensitive and functional biomarker for cellular stress. Owing to its rapid turnover, high susceptibility to degradation, and greater structural flexibility (*3*, *29*), RNA reflects metabolic perturbations earlier than DNA. Coupling with morphological analysis of the nucleus through different NIR channels enables multimodal readouts of complex cell states.

From a fundamental viewpoint, our study marks an important milestone in the development of multifunctional fluorophores capable of analyzing distinct biological processes with molecular precision. From a practical application perspective, **TEG**_**8**_**-N14** simplifies imaging workflows by reducing the need for multiple control groups, minimizing cytotoxicity, and avoiding UV-vis excitation. These features make it particularly suited for applications in live-cell assays and high-throughput drug screening. Looking ahead, advancing the understanding of pyrazinacene structure-function relationships and how they relate to biological phenomena is expected to drive substantial progress in both scientific research, industrial, and medical applications. Broadening the scope of **TEG**_**8**_**-N14** and its derivatives to in vivo systems could open previously unknown frontiers in single-cell diagnostics, spatiotemporal mapping of cell fate transitions, and preclinical evaluation of therapeutic efficacy.

## MATERIALS AND METHODS

### Synthesis of the precursor

1,2-Bis(3,4-bis(2-(2-(2-methoxyethoxy)ethoxy)ethoxy)phenyl)ethane-1,2-dione was prepared according to a literature method (*46*). Further synthesis scheme and details are provided in the Supplementary Materials.

### Photophysical properties

UV-vis absorption spectra were obtained using a UV-vis-NIR spectrometer (V-570) with a 0.1-nm resolution. Fluorescence spectra were recorded using a JASCO FP-6500 spectrometer with a microplate reader (Infinite 200 PRO, Tecan, Switzerland). All optical measurements were conducted using a 1.0-cm square quartz cuvette or black-bottom 96-well (Corning, 3991) plates. For fluorescence measurements, the sample solutions (2 μM) were excited at the specific absorption wavelength of 690 nm for each compound. The maximum absorbance values were plotted against concentration. The dye concentration for subsequent experiments was determined using the linear function previously plotted to ensure high reproducibility results for further experiments. Fluorescence quantum yields were determined using either a Hamamatsu Photonics C9920-02G absolute photoluminescence quantum yield spectrometer or a Hamamatsu Photonics Quantauras-QY absolute quantum yield spectrometer C11347-11. Quantum yields were measured in triplicate with rhodamine 6G as a reference, and the values were averaged.

### Fluorescence titration

Fluorescence titration experiments were conducted using calf thymus dsDNA and ssDNA (Wako Pure Chemical Industries, Japan), as well as RNA from torula yeast (Sigma-Aldrich Co., Ltd.), which were freshly annealed or dissolved in biotechnology-grade tris-EDTA buffer (Nacalai Tesque, Japan). All chemicals were used as received without further purification. Unless otherwise specified, black-bottom 96-well (Corning, 3991) plates were used to measure fluorescence intensities. FI spectra of **TEG**_**8**_**-N14** (2 μM) with stepwise addition of freshly prepared dsDNA, ssDNA, or RNA solution were measured with a microplate reader equipped with a monochromator and filters. The mixture was sealed with a sterile adhesive film, covered with aluminum foil, and gently shaken for 15 min to ensure homogeneity and stabilize the fluorescence intensities of all samples. Excitation and emission spectra were collected under maximum excitation and emission wavelengths under optimized conditions. Similar experiments were done by freshly preparing the **TEG**_**8**_**-N14** (2 μM) solutions with four different Na_2_HPO_4_-NaH_2_PO_4_ buffers (0.1 M, pH 5.8, 6.8, 7.4, and 8.0) and different storage temperatures (4°, 25°, and 37°C) for 1 day. All reagents and instruments used were biotechnology grade and checked for integrity, and the spectral data were baseline corrected. The reported data are representative results from multiple measurements.

### Cell culture and cell lines

HeLa [American Type Culture Collection (ATCC) CCL-2], NIH-3T3 (ATCC CRL-1658.2), and RAW Blue (CVCL_X594, Invivo Gen, San Diego, CA) cells were provided by M. Ebara (University of Tsukuba, Japan). B16F10 (ATCC CRL-6475) and ARPE-19 (ATCC CRL-2302) cells were purchased from ATCC. HeLa, NIH-3T3, RAW Blue, and B16F10 cells were maintained in high-glucose Dulbecco’s modified Eagle’s medium (DMEM) supplemented with 10% heat-inactivated fetal bovine serum (FBS; Gibco, Brazil) and 1% penicillin/streptomycin (Sigma-Aldrich Co., Ltd.) at 37°C with 5% CO_2_. ARPE-19 cells were maintained in DMEM:F-12 medium (ATCC 30-2006) supplemented with 10% FBS and 1% penicillin/streptomycin maintained in an incubator with a continuous supply of 5% CO_2_ at 37°C. The medium was refreshed every 2 days, and cells were split before reaching 80 to 90% confluency. The cell concentration for plating was assessed by adding trypan blue and measured using an automated cell counter (TC20, Bio-Rad Laboratories) and cell counting chambers. All reagents added to the cells were maintained mycoplasma-free under strictly aseptic conditions to avoid any contamination. The cell lines were routinely checked for mycoplasma contamination and confirmed to be mycoplasma-free.

### Cell toxicity based on cell number assay

NIH-3T3 cells (2.5 × 10^4^ cells) were seeded in the center of 24-well clear flat-bottom plates that had been coated with fibronectin (10 μg ml^−1^) and incubated overnight. After treatment with **TEG**_**8**_**-N14** (0 to 20 μM), the cells were trypsinized, and trypan blue was added to quantify the cell number using an automated counter. For cell counting, the results were also presented as percentages relative to the mean control.

### Cell dehydrogenase assay

NIH-3T3 cells were seeded at a density of 1 × 10^4^ cells per well in the center of 96-well clear flat-bottom plates (Iwaki, 3860-096) and cultured overnight. The cells were treated with **TEG**_**8**_**-N14** (0 to 20 μM) and AO (working concentration to stain DNA-RNA simultaneously: 200 μM) with and without FBS using a twofold serial dilution series and incubated for 24 hours. After incubation, cell metabolic activity was quantified according to the manufacturer’s instructions (Cell Counting Kit-8, Dojindo Molecular Technologies, Rockville, MD). Cell dehydrogenase activity was calculated using the following formula

$$\text{Cell dehydrogenase activity}\;\left(\%\right)=\frac{A_{\text{sample}}-A_{\text{blank}}}{A_{\text{control}}-A_{\text{blank}}}\times 100$$ (1)

where *A*_sample_ is the mean absorbance in wells containing a specific reagent concentration, *A*_control_ is the mean absorbance in wells with cells only, and *A*_blank_ is the mean absorbance in wells containing only solvents.

### ATP assay

NIH-3T3 cells (1 × 10^4^ cells) were resuspended in fresh culture medium and plated in the center of 96-well clear flat-bottom plates overnight. The cells were treated with the indicated compounds in a two-point twofold dilution series (200-μl final volume) and incubated for 24 hours. After equilibration to room temperature, the diluted Cell Titer-Glo 2.0 luminescence-based assay (Promega, Japan) was added to the cells under aseptic conditions to avoid adenosine 5′-triphosphate (ATP) contamination. The supernatant was transferred into sterile 96-well white flat-bottom microplates (Greiner Bio-One, 655904), and the plates were sealed with an adhesive film, covered with aluminum foil, and shaken on an orbital shaker for 10 min before luminescence measurement using an Ensight (Perkin Elmer, Japan) plate reader. The data acquired from ATP assays were validated using an ATP standard curve to ensure their integrity. The data obtained were normalized to the average control and presented as percentages after subtracting the absorbance from solvent-only wells.

### Fixation

One day before imaging, cells were seeded onto a 35-mm glass-bottom dish (Greiner Bio, 627870) and cultured overnight. The cells were washed once with ice-cold phosphate-buffered saline (PBS), prefixed in 4% paraformaldehyde, and permeabilized in 0.5% (v/v) Triton X-100 in PBS for 10 min. After washing three times in PBS, the cells were labeled with **TEG**_**8**_**-N14** (10 μM) with and without addition of CTAB and imaged in PBS as an imaging medium.

### DNA-RNA specificity assay

For DNA colocalization experiments, fixed HeLa cells (4 × 10^4^ cells) that had been stained with **TEG**_**8**_**-N14** (10 μM) and Hoechst 33342 (1 μM, 5 min) were used. After washing with ice-cold PBS, fluorescence images were obtained from three different channels, namely the blue channel (λ_ex_ = 405 nm and λ_em_ = 415 to 600 nm for Hoechst 33342), cyan channel (λ_ex_ = 640 nm and λ_em_ = 650 to 720 nm for **TEG**_**8**_**-N14**), and magenta channel (λ_ex_ = 730 nm and λ_em_ = 740 to 850 nm for **TEG**_**8**_**-N14**).

To quantitatively assess RNA-specific colocalization, nucleolus bright green (Dojindo, Japan) was used to counterstain **TEG**_**8**_**-N14**–stained cells. Images were captured using the purple channel (λ_ex_ = 450 nm and λ_em_ = 500 to 550 nm) and cyan channel (λ_ex_ = 640 nm and λ_em_ = 650 to 720 nm) after subtracting the background from each channel to eliminate potential bias in the results. For further RNA specificity analysis, after fixation and permeabilization of HeLa cells, the cells were washed with ice-cold PBS, digested with RNase A or DNase I (Nippon Gene, Japan) at 37°C for 30 min, washed with PBS, stained with **TEG**_**8**_**-N14**, and subsequently incubated in PBS. Images were acquired using the following channels: cyan channel (λ_ex_ = 640 nm and λ_em_ = 650 to 720 nm) and magenta channel (λ_ex_ = 730 nm and λ_em_ = 740 to 850 nm).

For the signal-to-background measurement and brightness comparison of **TEG**_**8**_**-N14**, fixed HeLa cells were imaged volumetrically using a similar microscope setup, and FI was subjected to a threshold. To check compatibility with different types of microscopes, an Olympus FV3000 was also used.

### Computational modelling

Molecular docking simulations were performed using the xTB program (*47*). The DNA 12-nucleotide oligomer [Protein Data Bank (PDB) accession code: 1D29] (*48*), ssRNA (PDB accession code: 5WWF; to avoid complications, RNA binding protein was removed before docking study with **TEG**_**8**_**-N14**) (*49*), and dsRNA (PDB accession code: 4MCE) (*50*) structures were selected from the PDB, optimized using the GFN2-xTB method (*51*) with an implicit water solvent model (ALPB) (*52*), and assigned a charge to account for the phosphate backbone. Similarly, the **TEG**_**8**_**-N14** molecules and N14 core model structures were optimized. Docking simulations between the optimized DNA dodecamer and the N14 model were conducted using the xTB docker module (*53*) at the GFN2-xTB level with the following parameters: rotational step size of 1°, translational step size of 10 pm, maximum of 20 generations in the genetic algorithm, parent population size of 200, and 30 final structures. All calculations used the LBFGS optimization engine with 10 maximum cycles and 10 microcycles per geometry optimization step. The final structures were then analyzed to consider the distances between the N14 center of mass and the nucleotide structural motifs.

### Confocal microscopy for cell imaging

Confocal images were captured using a custom-built STELLARIS 8 DIVE microscope (Leica Microsystems) equipped with a white light laser offering tunable excitation wavelengths ranging from 440 to 790 nm and operating at ~80 MHz. For subsequent cell imaging experiments, excitation wavelengths of 640 and 730 nm were selected for imaging cell samples stained with **TEG**_**8**_**-N14**. Spectral detection was carried out using calibrated Power HyD X photon-counting detectors within the spectral ranges of 650 to 720 nm and 740 to 850 nm. For low- and high-magnification imaging, an HC PL APO CS2 40×/1.1 water immersion objective and HC PL APO CS2 63×/1.40 oil immersion objective were used, respectively. The acquired confocal images were background subtracted, leading to acquired images with pixel array dimensions of 1024 by 1024 with 1.0 Airy units. Lightning mode was used to obtain a higher resolution of images. All samples were equilibrated at 37°C for 30 min to prevent thermal drift during image acquisition.

### Two-dimensional spectral cell imaging

Fixed HeLa cells (4 × 10^4^ cells per compartment) that had been stained with **TEG**_**8**_**-N14** with or without CTAB were imaged with fluorescence spectra also being captured using STELLARIS 8 with excitation in the range of 440 to 790 nm at a bandwidth of 10 nm. Fluorescence spectral detection was performed with Power HyD X photon-counting detectors using 3-nm spectral steps in the window of 450 to 820 nm at a bandwidth of 10 nm. Images were acquired using an HC PL APO CS2 63×/1.40 oil immersion objective (Leica Microsystems). To avoid bias in the results, the laser power, gain, and dwell time were maintained almost constant and optimized before actual measurements. For brightness comparison, *z*-stack images of cells were captured using a similar microscopy setup with a step size of ~1 μm and ~22-μm depth. A threshold was applied to the FI, and it was analyzed within the cells.

### Costaining for multicolor imaging application

HeLa cells were costained with **TEG**_**8**_**-N14** in the presence of CTAB (2.5 μM) with various commercial dyes according to the manufacturers’ instructions with unmixing linear spectra. The image channels used were described as follows: i-Phalloidin (λ_ex_ = 405 nm and λ_em_ = 415 to 445 nm); magenta: Lipi Green (λ_ex_ = 470 nm and λ_em_ = 500 to 600 nm) or Lyso Green (λ_ex_ = 450 nm and λ_em_ = 500 to 600 nm); green: **TEG**_**8**_**-N14** (λ_ex_ = 640 nm and λ_em_ = 650 to 720 nm); purple: **TEG**_**8**_**-N14** (λ_ex_ = 730 nm and λ_em_ = 740 to 850 nm). To further examine its specificity over other biomolecules, the cell lysate was extracted from HeLa cells using a cell lysis buffer (Cell Signaling Technology, Japan) according to the manufacturer’s instructions and then digested with DNase I and RNase A at 37°C for 24 hours. Next, **TEG**_**8**_**-N14** was added, and the mixture was allowed to stabilize for 15 min. Fluorescence spectra were then quantified using a microplate reader (Infinite 200 PRO, Tecan, Switzerland).

### Photostability assay

After HeLa cells had been fixed and stained with **TEG**_**8**_**-N14** in the presence of CTAB (2.5 μM, 15 min), SYTO 63 (500 nM, 30 min), MitoTracker Deep Red (100 nM, 30 min), SiR640-DNA (50 nM, 30 min), and SiR700-DNA 700 nm (50 nM, 30 min), cell images were continuously photobleached using an oil-immersion objective lens (HC PL APO CS2 63×/1.4 Oil). Laser lines (excitation at 640 or 730 nm) were adjusted to an output power, measured with a calibrated power meter (PM400, Thorlabs Inc.) equipped with a calibrated microscope slide power meter sensor (S170c, Thorlabs Inc.). Fluorescence signals were recorded at ×4 magnification for the region of interest (2125.3 μm^2^; 46.1 by 46.1 μm). The confocal aperture size was 1 Airy disk, and the voxel size was 0.5 by 0.5 μm at a scan speed of 400 Hz. Nuclei of similar sizes and intensity were selected for the photobleaching assays under an identical instrument setup. Fluorescence signals were detected over the emission wavelength range of 650 to 670 nm or 740 to 850 nm using a calibrated laser and Power HyD X photon-counting detectors, and the best-focus frame of 10 series images was used to measure fluorescence signal intensity. The photobleaching half-time was determined as the time taken to decrease the scan-averaged emission rate to 50% from an initial rate of 1000 photons/s per molecule based on a reported method (*14*). The average photon flux [photons/(s·m^2^)] over the scanned area of interest was calculated as follows

$$\Phi ==\frac{P}{A}\cdot \frac{1}{\left(\frac{\mathrm{hc}}{\lambda }\right)}$$ (2)

where *P* is the output power of the laser measured at the sample plane in W, *A* is the area of the view field in cm^2^, and $\frac{\mathrm{hc}}{\lambda }$ is the energy of a photon at the certain laser wavelength (640 or 730 nm). The optical cross section in cm^2^ was calculated as follows

$$\sigma \;\left(\lambda \right)=\frac{\left(1000\;{\text{cm}}^{3}/\text{liter}\right)\left(\text{ln}10\right)\varepsilon \left(\lambda \right)}{6.023\times 1023/\text{mole}}$$ (3)

where ε(λ) is the extinction coefficient of the dye at the laser wavelength, and the scan average excitation rate per fluorescent molecule was calculated by using

x=Φσ(λ) (4)

Bleaching time was calculated on the basis a decrease in the scan-averaged rate from 1000 to 500 photons/s as follows

$$t_{1/2}=\frac{t_{\mathrm{raw}}XQ}{1000\;\text{photon}/s}$$ (5)

To generate complete bleaching curves, we first converted the raw time data into the number of excitations per molecule using the factor $\frac{XQ}{1000\;\text{photon}/s}$ and normalized the intensity axis to an initial emission rate of 1000 photons/s (*14*).

### Imaging of different states of cell injury in fixed NIH-3T3 cells

Before cell treatment, 35-mm glass-bottom dishes or the well centers of 24-well black glass-bottom plates (Greiner Bio-One, 662892) were coated with fibronectin (10 μg ml^−1^) and equilibrated in an incubator at 37°C for 2 hours. After washing with prewarmed PBS, NIH-3T3 fibroblasts were seeded at a density of 1.0 × 10^4^ cells and cultured at 37°C with 5% CO_2_ for 24 hours. Before imaging, the cells were treated with staurosporine (1 μM), H_2_O_2_ (1 mM), and TNF-α (100 ng mL^−1^) + Z-VAD-FMK (20 μM) for 2, 6, and 24 hours. For PI staining, the cells were washed, incubated with dye according to the manufacturer’s instructions, and further imaged in phenol red–free DMEM. For **TEG**_**8**_**-N14** staining, the cells were fixed, stained, and incubated in PBS, followed by *z*-stack imaging conducted with ~1-μm steps using an optimized microscope setup during measurement. For FI quantification measurement, all images were quantified using identical settings with a fluorescence threshold (Otsu method). The summed stacks were analyzed as follows: The mean intensity of a rectangular region of interest within the treated cell was normalized to the average intensity of an untreated cell. For segmentation based on morphology, images obtained from the cyan channel (λ_ex_ = 640 nm and λ_em_ = 650 to 720 nm) and magenta channel (λ_ex_ = 730 nm and λ_em_ = 740 to 850 nm) were each categorized into three groups and quantified by a blinded observer using randomly selected images with different fields of view.

### Imaging of different states of cell injury in live NIH-3T3 cells

Briefly, NIH-3T3 fibroblasts (1.0 × 10^4^ cells) were seeded on fibronectin-coated glass-bottom dishes and further incubated at 37°C with 5% CO_2_ for 24 hours. Before imaging, the cells were treated with staurosporine (1 μM), H_2_O_2_ (high concentration, 1 mM), and H_2_O_2_ (low concentration, 200 μM) for 2, 24, or 36 hours to induce apoptosis, necrosis, or mixed apoptosis/necrosis, respectively. After a 30-min incubation with **TEG**_**8**_**-N14** (10 μM), the cells were imaged using a microscope equipped with a controllable incubator for CO_2_ and temperature. A positive control combining single DNA (Hoechst 33342) and RNA dyes (Nucleolus Bright Green, Dojindo, Japan) was prepared to investigate potential differences compared to **TEG**_**8**_**-N14**.

### Imaging of different cell senescence induction

ARPE-19 cells with different passage numbers were imaged under a confocal microscope under different excitation and emission conditions (405/415 to 460, 488/500 to 550, 561/571 to 635, 640/650 to 720, and 730/740 to 850 nm). Subsequently, ARPE-19 cells were seeded in a 35-mm glass-bottom dish, and Dox (250 nM) was added consecutively to the cells on each of the three following days. Subsequently, normal and senescent cells were fixed, stained, and imaged at different time points using an optimized microscope setup to avoid bias in the results. To ensure that most cells underwent senescence, a positive control was prepared and the number of untreated and treated cells was counted using an automated cell counter. For comparison, ARPE-19 cells alone were used or SPIDER β-Gal (Dojindo, Japan) was used to stain the cells according to the manufacturer’s instructions, and all images were baseline corrected. The samples were observed at the different excitation and emission wavelengths mentioned above.

### Statistical analysis and reproducibility

Representative confocal images are displayed. Each experiment was conducted on at least two independent experiments, unless otherwise noted. The different *P* values are presented as *P < 0.05, **P < 0.01, and ***P < 0.001, determined using an unpaired two-tailed Student’s *t* test; n.s. means a notable signal. Statistical analysis and curve fitting were conducted using Microsoft Excel 2019 or Origin Pro 2022. For quantitative analysis, all raw data without further processing were quantified using ImageJ/Fiji, Imaris, and LAS X software (Leica Microsystems). To avoid bias in the results, all images underwent background subtraction with a positive control, fluorescence was subject to a threshold using a global method (Otsu method), and measurements were randomly taken from volumetric imaging across different fields of view, excluding the edges. For representative confocal images, fluorescence images were acquired using lightning mode, multiplane images were converted into maximum intensity projections, and representative confocal images were finally divided into 5 by 5–pixel kernels.
