# Supplementary material for: Visualizing the chronicle of multiple cell fates using a near-IR dual-RNA/DNA–targeting probe
Source: Sci Adv. 2025 Oct 22;11(43):eadz6633. doi: 10.1126/sciadv.adz6633 (PMC12542947; doi:10.1126/sciadv.adz6633)
Supplement: Supplementary file 1 — Data S1 Figs. S1 to S41 Tables S1 and S2 Legends for movies S1 to S4 [file sciadv.adz6633_sm.pdf]

Science Advances
AAAS

Supplementary Materials for

Visualizing the chronicle of multiple cell fates using a near-IR dual-RNA/DNA–targeting probe

Linawati Sutrisno *et al.*

Corresponding author: Linawati Sutrisno, sutrisno.linawati@nims.go.jp; Jonathan P. Hill, jonathan.hill@nims.go.jp; Masayasu Taki, taki.masayasu.s8@f.gifu-u.ac.jp; Katsuhiko Ariga, ariga.katsuhiko@nims.go.jp

Sci. Adv. **11**, eadz6633 (2025)
DOI: 10.1126/sciadv.adz6633

The PDF file includes:

Data S1
Figs. S1 to S41
Tables S1 and S2
Legends for movies S1 to S4

Other Supplementary Material for this manuscript includes the following:

Movies S1 to S4

Supplementary Materials

Data S1

General: Reagents, dehydrated solvents (in septum-sealed bottles) and buffer solutions used for syntheses, cell culturing and spectroscopic measurements were obtained from Tokyo Kasei Chemical Co., Wako Fujifilm Chemical Co., Nacalai Tesque Chemical Co. or Sigma-Aldrich Chemical Co. and were used without further purification. Electronic absorption spectra were measured using JASCO V-570 UV/Vis/NIR and JASCO V770 UV/Vis/NIR spectrophotometers with a 400 nm/min scan rate. FTIR spectra were obtained using a Shimadzu FTIR-8400S spectrophotometer from samples prepared as KBr discs. ^{1}H-NMR spectra were obtained using a JEOL JNM-ECZL400S spectrometer operating at 400 MHz. Proton decoupled ^{13}C-NMR spectra were obtained using a JEOL AL400SSS spectrometer operating at 101 MHz. ^{1}H NMR and ^{13}C NMR chemical shifts (δ) are reported in ppm relative to the residual solvent peak. NMR spectra were processed using the MestReNova software package. MALDI-TOF mass spectra were measured using a Bruker Daltonics autoflex maX mass spectrometer with dithranol as matrix. High resolution electrospray ionization time-of-flight mass spectra (ESI-TOF-MS) were measured using a Thermo Scientific Q-Exactive Plus instrument. 1,2-Bis(3,4-bis(2-(2-(2-methoxyethoxy)ethoxy)ethoxy)phenyl)ethane-1,2-dione was prepared according to a literature method (*46*).

Synthesis of 6,7-Bis(3,4-bis(2-(2-(2-methoxyethoxy)ethoxy)ethoxy)phenyl)pyrazino[2,3-*b*]pyrazine-2,3-dicarbonitrile, TEG$_4$-TAN(CN)$_2$

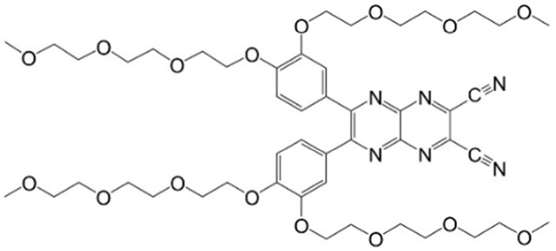

Chemical structure of **TEG$_4$-TAN(CN)$_2$**

A mixture of 1,2-bis(3,4-bis(2-(2-(2-methoxyethoxy)ethoxy)ethoxy)phenyl)ethane-1,2-dione (3.5 g, 4.07 × 10^{-3} mol), 5,6-diamino-2,3-dicyanopyrazine (0.98 g, 6.13 × 10^{-3} mol) and *p*-toluenesulfonic acid monohydrate (150 mg, 7.89 ×10^{-4} mol) in dry toluene (150 mL) in a 300 mL flask equipped with a Dean-Stark trap was heated at reflux for 4 days. The reaction mixture was then allowed to cool to room temperature, the solution was filtered, and the filtrate concentrated yielding a dark purple oil. The crude product was then purified sequentially by using column chromatography (SiO_2; gradient elution: 100% CH_2Cl_2 to 2.5% MeOH in CH_2Cl_2) followed by gel permeation chromatography (Bio-Rad Bio-Beads S-X1 support, CH_2Cl_2) to yield the product as a dark red oil. Yield: 3.3 g (82 %). ^{1}H NMR (600 MHz, $CDCl_3$, 18 °C) δ = 7.55 (d, J = 2.2 Hz, 2H, ArH), 7.23 (dd, J = 8.5, 2.1 Hz, 2H, ArH), 6.81 (d, J = 8.2 Hz, 2H, ArH), 4.21 (t, J = 4.5 Hz, 4H, OCH_2), 4.15 (t, J = 4.9 Hz, 4H, OCH_2), 3.90 (t, J = 4.9 Hz, 4H, OCH$_2$CH_2), 3.87 (t, J = 4.9 Hz, 4H, OCH$_2$CH_2), 3.77-3.73 (mult, 8H, OCH_2), 3.69-3.63 (mult, 16H OCH_2CH_2), 3.56-3.53 (mult, 8H, OCH$_2$CH_2), 3.37 (s, 6H, OCH_3), 3.37 (s, 6H, OCH_3) ppm. ^{13}C NMR (150 MHz, $CDCl_3$, 18 °C) δ = 161.5, 152.9, 149.2, 143.7, 132.6, 129.2, 125.5, 115.3, 113.0, 112.7, 72.0, 71.1, 71.0, 70.8,

70.7, 69.7, 69.5, 69.0, 68.7, 59.2 ppm. FTIR (KBr pellet): ν = 2930 (s), 2884 (s, C-H str.), 1599 (m, C=C str.), 1516 (m, C=N str.), 1425 (m, C-H def.), 1375 (s, C-H def.), 1333 (m, C-H def.), 1269 (s, C-O asymm. str.), 1136 (s, C-O symm. str.), 1053 (m), 949 (m), 854 (w), 615 (w), 550 (w), 502 (w) cm^{-1}. MALDI-TOF-MS (-ve mode, dithranol): Calcd for $C_{48}H_{67}N_6O_{16}$ ($[M + H]^-$): 983.461, found: 983.466 amu.

Synthesis of 2-Perdeuteriomethoxy-6,7-bis(3,4-bis(2-(2-(2-methoxyethoxy)ethoxy)ethoxy)phenyl)pyrazino[2,3-*b*]pyrazine-3-carbonitrile

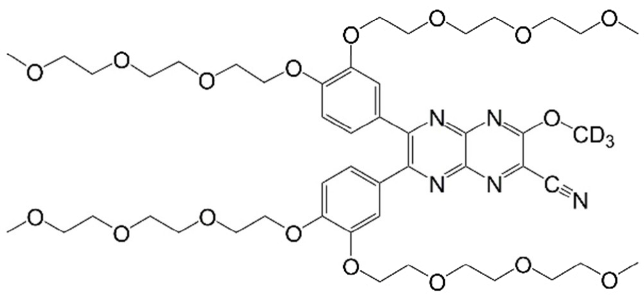

Chemical structure of **TEG_4-TAN(CN)$_2$**, where one nitrile reacts with CD_3OD upon warming

6,7-Bis(3,4-bis(2-(2-(2-methoxyethoxy)ethoxy)ethoxy)phenyl)pyrazino[2,3-*b*]pyrazine-2,3-di carbonitrile (5 mg, 5.1×10^{-6} mol) was dissolved in tetrahydrofuran-d_8 (0.8 mL) in a standard sample tube (⌀ 5 mm) for NMR spectroscopy and CD_3OD (200 μL) was added. The solution was warmed overnight (16 h) at 50 °C then ^{1}H-NMR, ^{13}C-NMR and other analyses were carried out to identify the product. ^{1}H NMR analysis indicates almost quantitative conversion to 2-perdeuteriomethoxy-6,7-bis(3,4-bis(2-(2-(2-methoxyethoxy)ethoxy)ethoxy)phenyl)pyrazino [2,3-*b*]pyrazine-3-carbonitrile. ^{1}H NMR (400 MHz, THF-d_8/CD_3OD, 18 °C) $\delta = 7.37$ (d, $J = 2$ Hz, 1H, Ar*H*), 7.32 (d, $J = 2.4$ Hz, 1H, Ar*H*), 7.23 (dd, $J_1 = 8.8$ Hz, $J_2 = 2.4$ Hz, 1H, Ar*H*), 7.16 (dd, $J_1 = 8.0$ Hz, $J_2 = 2.0$ Hz, 1H, Ar*H*), 6.96 (d, J = 7.6 Hz, 2H, Ar*H*), 4.17 (t, $J = 3.6$ Hz, 4H, Ar-OCH_2-), 4.04 (m, 4H, Ar-OCH_2-), 3.83 (t, $J = 5.2$ Hz, 4H, Ar-OCH$_2$CH_2-O), 3.73 (m, 4H, Ar-OCH$_2$CH_2-O), 3.68 (m, 4H, -CH_2-), 3.56 (m, overlaps solvent peak), 3.46 (m, 8H, -CH_2-), 3.29 (s, 6H, -O-CH_3), 3.28 (s, 6H, -O-CH_3) ppm. ^{13}C NMR (100 MHz, $CDCl_3$, 18 °C): $\delta = 159.84$, 158.18, 154.75, 151.39, 150.81, 148.82, 143.33, 140.04, 130.89, 130.63, 124.93, 124.21, 123.60, 116.35, 116.14, 113.59, 113.37, 113.10, 71.90, 70.74, 70.67, 70.55, 70.32, 69.49, 69.46, 69.39, 68.96, 68.78, 68.74 ppm. MALDI-TOF-MS (-ve mode, dithranol): Calcd for $C_{48}H_{66}D_3N_5O_{17}$ ($[M + H]^-$): 991.496, found: 991.649 amu.

Synthesis of 2,3,11,12-Tetrakis(3,4-bis(2-(2-(2-methoxyethoxy)ethoxy)ethoxy)phenyl)-7,16-dihydro-1,4,5,6,7,8,9, 10,13,14,15,16,17,18-tetradecaazaheptacene

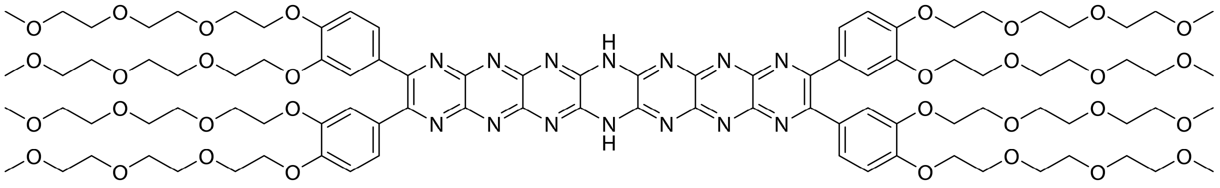

Chemical structure of **TEG_4-N14**

A mixture of freshly prepared 2,3,5,6-pyrazinetetramine (20 mg, 1.43×10^{-4} mol), super dehydrated dimethyl sulfoxide (1.5 mL) and freshly ground potassium carbonate (200 mg, 1.45×10^{-3} mol) was heated at 122 °C for 10 minutes. The reaction mixture was flushed with air and a solution of 6,7-bis(3,4-bis(2-(2-(2-methoxyethoxy)ethoxy)ethoxy)phenyl)pyrazino[2,3-*b*]pyrazine-2,3-dicarbonitrile (418 mg, 4.25×10^{-4} mol) in DMSO (3 mL) was added to the reaction mixture followed by additional potassium carbonate (2.16 g, 1.57×10^{-2} mol). The reaction mixture was again briefly flushed with air followed by stirring at 122 °C for 1 hour. After cooling to room temperature, DMSO was removed under reduced pressure to obtain a solid residue that was partitioned between chloroform (100 mL) and aqueous ammonium chloride (2.5 g, 4.7×10^{-2} mol in deionized water (30 mL)). The aqueous layer was separated, extracted with chloroform (2 × 20 mL), then organic layers were combined and dried over sodium sulfate. After filtration, the solvent was removed under reduced pressure. The crude product was purified by repeated column chromatography procedures. A preliminary column (SiO_2; gradient elution: 100% $CHCl_3$ to 92:6:2 $CHCl_3$/MeOH/Et_3N]. was followed by preparative GPC (Bio-Rad Bio-Beads SX-3, $CHCl_3$), column chromatography twice (SiO_2; gradient elution 100% $CHCl_3$ to 96:2:2 $CHCl_3$/MeOH/Et_3N). A final step of purification was made using PTLC (SiO_2, 88:12 $CHCl_3$/MeOH]. The product was dissolved in $CHCl_3$ (2 mL) and washed with saturated ammonium chloride solution (3 × 3 cm^3), brine (3 cm^3) and dried over sodium sulfate. The mixture was filtered and the filtered solid concentrated to a blue solid. Yield 30 mg (11 %). ^1H NMR (400 MHz, DMSO-d_6, 75 °C): δ = 7.22 (d, J = 2.1 Hz, 4H, Ar*H*), 7.20 (dd, J = 8.2, 2.1 Hz, 4H, Ar*H*), 7.06 (d, J = 8.3 Hz, 4H, Ar*H*), 4.18 (t, J = 5.3 Hz, 8H, OCH_2), 4.00 (t, J = 4.3 Hz, 8H, OCH_2), 3.79 (t, J = 4.5 Hz, 8H, OCH_2CH_2), 3.68 (t, J = 4.6 Hz, 8H, OCH_2CH_2), 3.66-3.50 (mult, 48H, OCH_2CH_2) 3.48-3.42 (mult, 16H, OCH_2CH_2), 3.27 (s, 12H, OCH_3), 3.24 (s, 12H, OCH_3) ppm. ^{13}C NMR (150 MHz, THF-d_8/1 drop TFA-*d*, 65 °C): δ = 155.9, 150.8, 148.6, 146.09, 145.31, 143.88, 131.37, 123.83, 116.58, 113.54, 71.69, 70.50, 70.43, 70.37, 70.33, 70.26, 70.13, 70.10, 69.42, 69.34, 69.28, 69.19, 68.98, 68.86, 68.78, 57.62 ppm. FTIR (KBr pellet): ν = 3431 (br, s), 2916 (s), 2890 (s), 1597 (m), 1516 (s), 1421 (s), 1368 (s), 1327 (s), 1258 (s), 1182 (s), 1117 (s), 1051 (m), 949 (m), 827 (w), 664 (w), 606 (w), 488 (w) cm^{-1}. ESI-MS (negative mode): calc'd for $C_{96}H_{133}N_{14}O_{32}$ $[M - H]^+$: 1993.9210, found: 1993.9208.

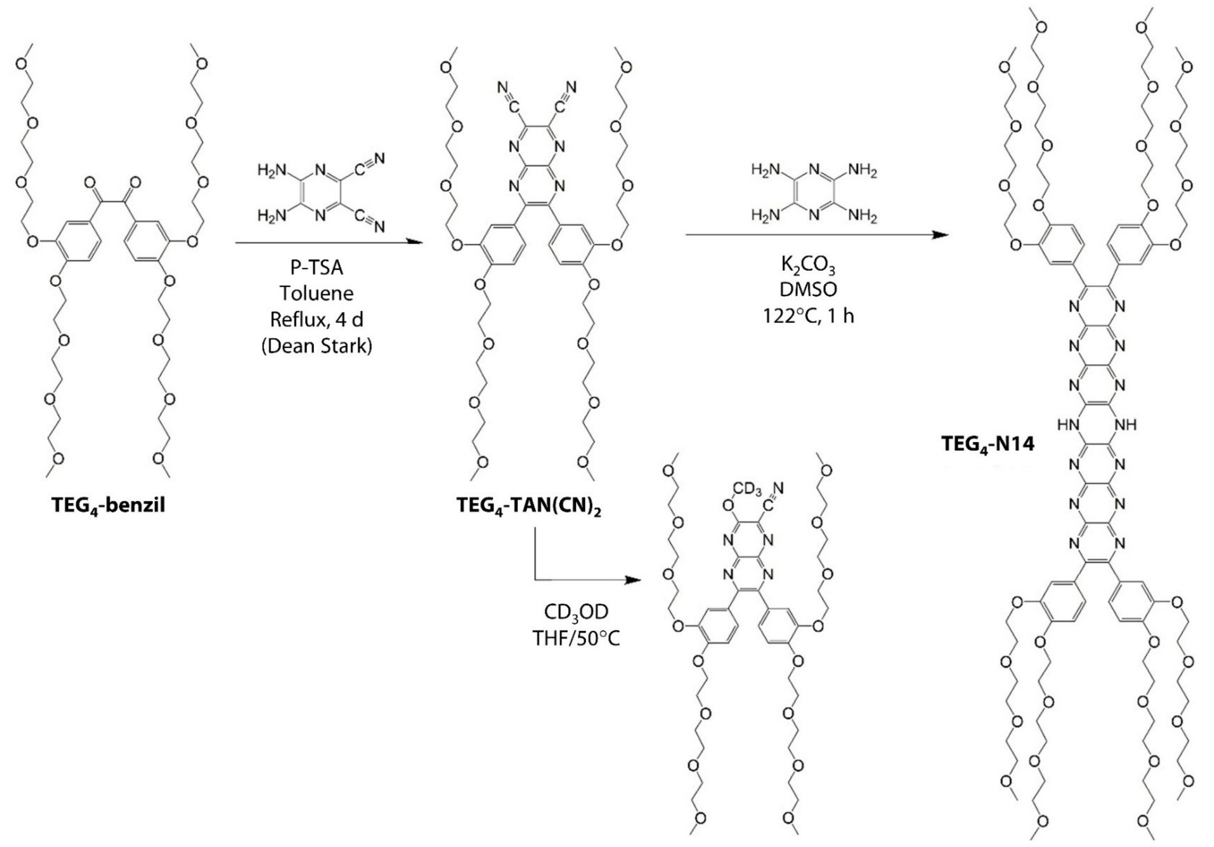

Fig. S1. **Synthetic scheme of water soluble TEG$_8$-N14**. **TEG$_4$-benzil** was condensed with 5,6-diaminopyrazine-2,3-dicarbonitrile catalyzed by p-toluenesulfonic acid in refluxing toluene in Dean-Stark apparatus. The resulting **TEG$_4$-TAN(CN)$_2$** was then condensed with pyrazine-2,3,5,6-tetramine yielding the target N14 compound 2,3,11,12-tetrakis(3,4-bis(2-(2-(2-methoxyethoxy)ethoxy)ethoxy)phenyl)-7,16-dihydro-1,4,5,6, 7,8,9,10,13,14,15,16,17,18-tetradecaazaheptacene. **TEG$_4$-TAN(CN)$_2$** is unstable against nucleophilic substitution so that one nitrile group reacts when warmed with, for example here, CD_3OD. This reaction accounts for an impurity contained in **TEG$_4$-TAN(CN)$_2$** but does not affect the subsequent reaction since addition of methanol deactivates the compound against reaction with phenylene-1,2-diamines.

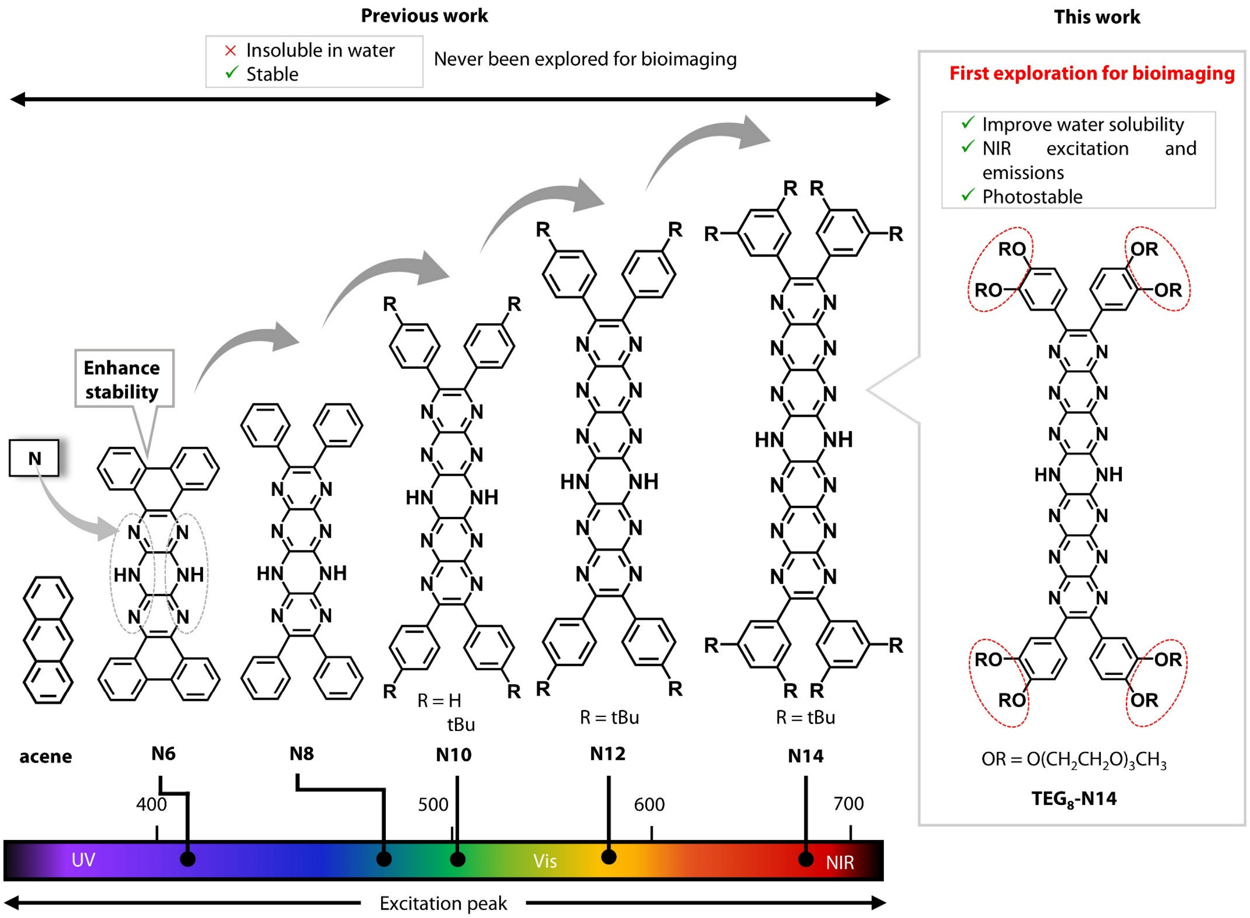

Fig S2. Design, synthesis and characterization of extended-pyrazinacene dyes. Previously prepared pyrazinacenes (left side) based on multiplicity of nitrogen atoms (*11*). In this study, a modified version of N14 synthesized with triethylene glycol monomethyl ether (TEG) substituents, TEG_8-N14, is presented. To promote disaggregation of TEG_8-N14, cetyltrimethylammonium bromide (CTAB) was introduced as a surfactant.

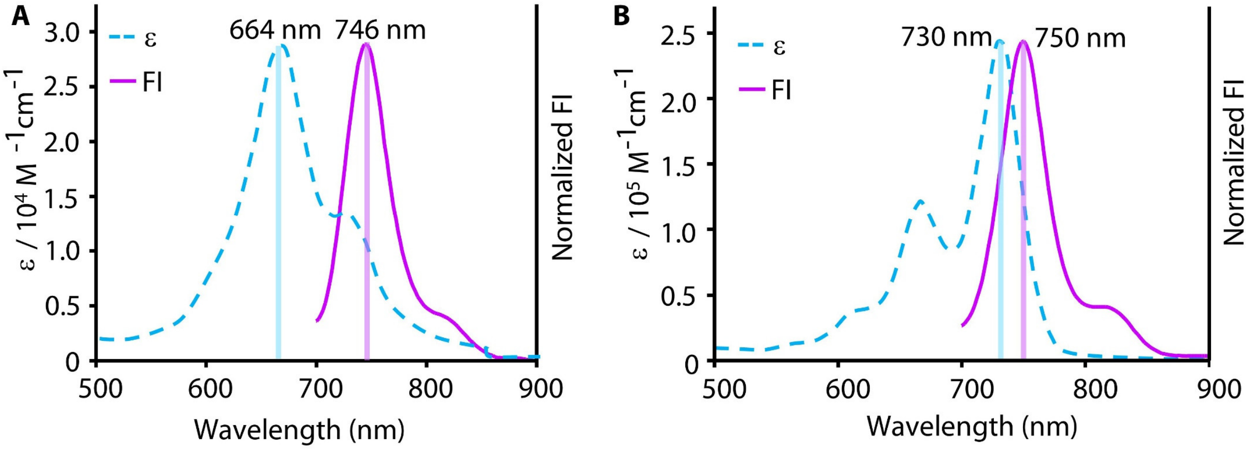

Fig. S3. Molar extinction coefficient and emission spectrum of TEG_8-N14 without and with addition of CTAB as surfactant. Molar absorptivity spectra (dash line) and normalized

fluorescence emission (λ_{ex} = 690 nm, solid line) spectra of TEG$_8$-N14 (left, **A**) and TEG$_8$-N14 with addition of CTAB (right, **B**) in PBS (10 mM, pH 7.4). All spectra are baseline corrected.

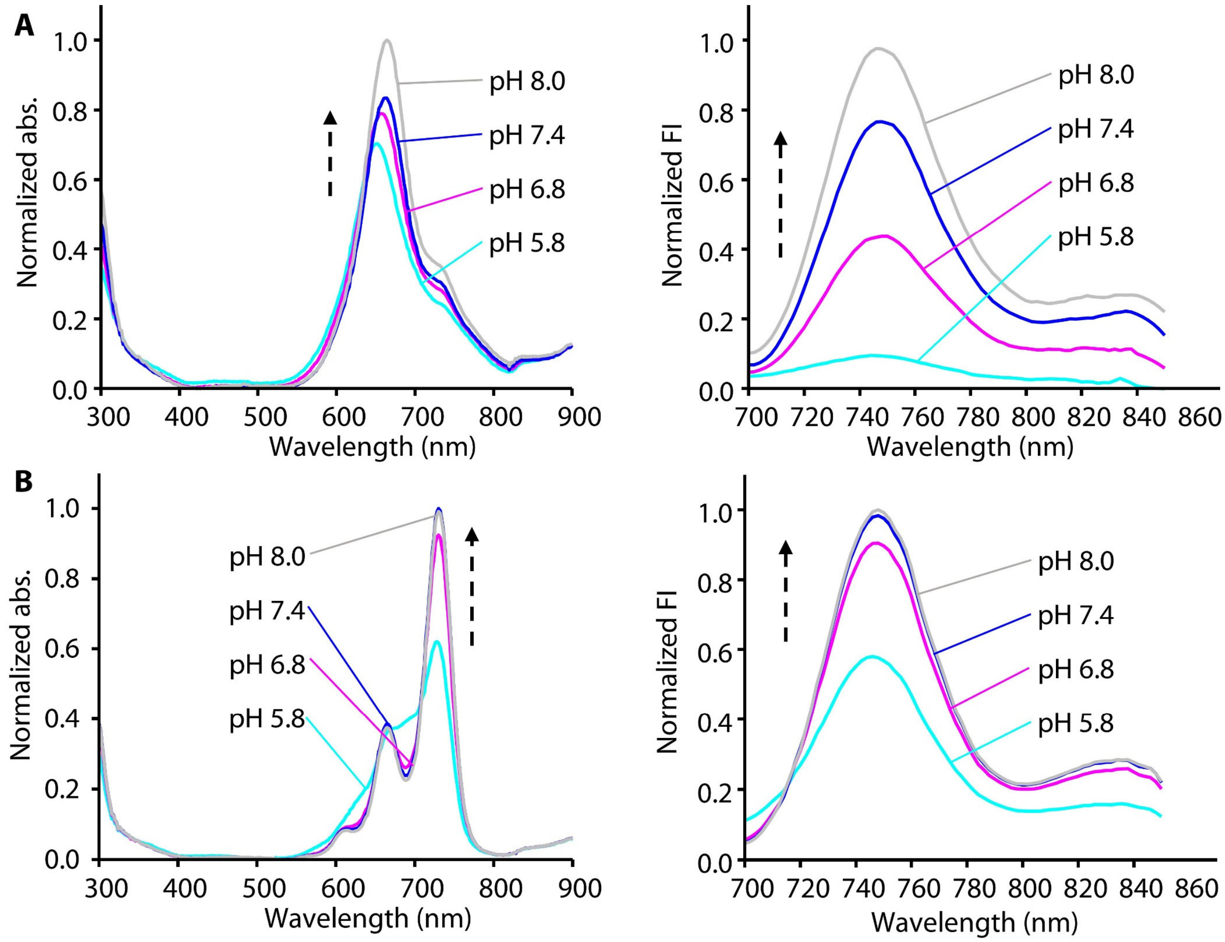

Fig. S4. Effect of pH. UV–vis absorption and emission spectra (λ_{ex} = 690 nm) of TEG$_8$-N14 (**A**) and TEG$_8$-N14 in the presence of CTAB (**B**) in four different Na_2HPO_4-NaH_2PO_4 buffers (0.1 M, pH 5.8, 6.8, 7.4, and 8.0). All spectra are baseline corrected.

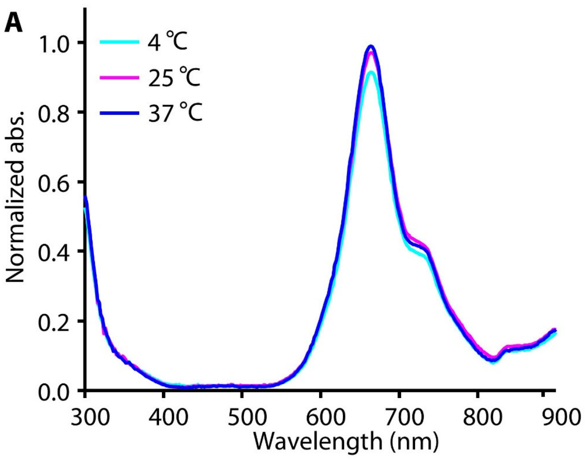

Fig. S5. Effect of temperature. Normalized UV–vis absorption spectra of TEG_8-N14 (**A**) and TEG_8-N14 in the presence of CTAB (**B**) with three different temperatures (4, 25, 37°C). All spectra are baseline corrected.

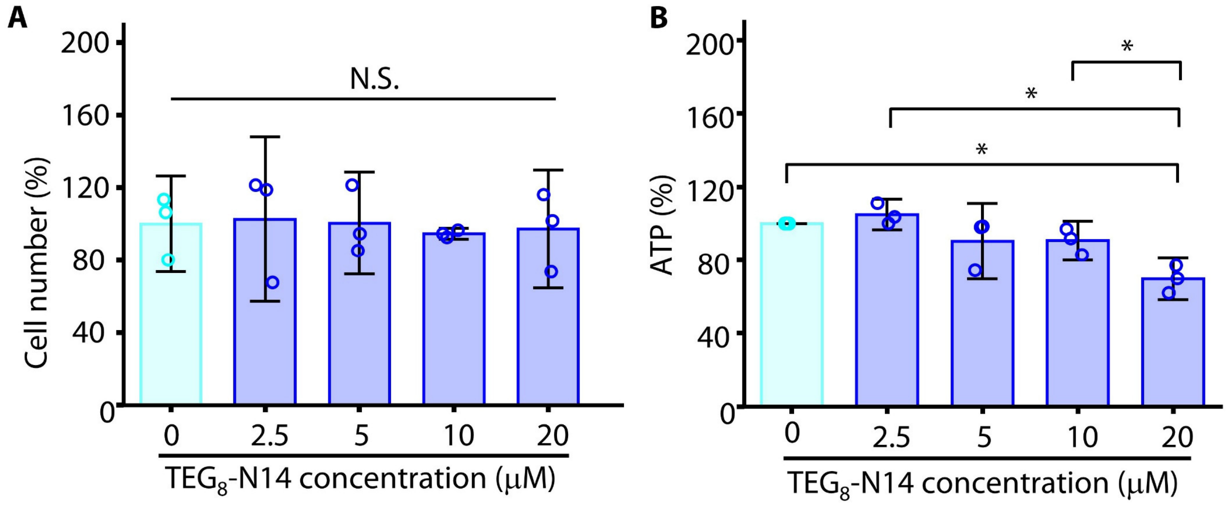

Fig. S6. Effect of probes on cellular toxicity. (**A**) Quantification of cell counts of untreated versus treated cells with different concentrations of TEG8-N14 using Trypan Blue assay. Data presented as mean ± S.D. (n=3) of three independent experiments. N.S.: notable signal. (**B**) Measurement of cellular energy with percentage intracellular ATP levels, mean ± S.D. (*n*=3), *p < 0.05 by two-tailed unpaired Student's *t*-test. ATP, adenosine triphosphate.

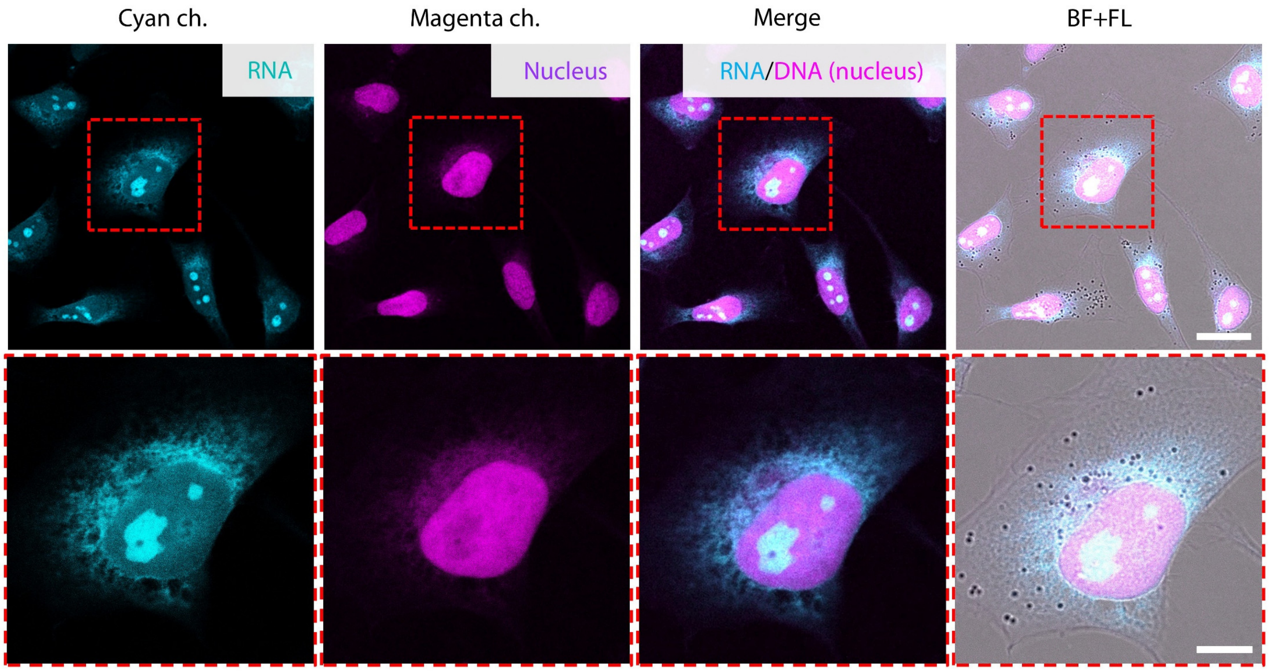

Fig. S7. RNA-DNA discrimination with single fluorophore in HeLa cells. Representative confocal images (*n*=3 from each experiment) of fixed HeLa cells labeled with TEG8-N14. Cyan channel (λ_{ex} = 640 nm and λ_{em} = 650 – 720 nm) and magenta channel (λ_{ex} = 730 nm and λ_{em} = 740 – 850 nm). Scale bars: 10 μm.

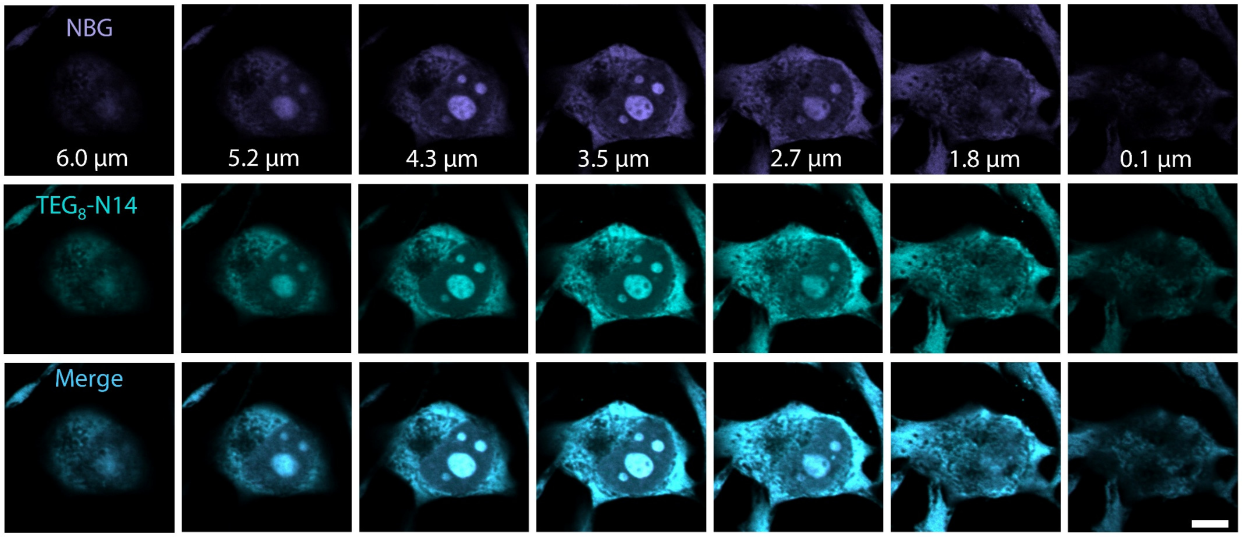

Fig. S8. RNA highly specific labeling TEG$_8$-N14 in fixed HeLa cells. Z-stacks colocalization imaging of HeLa cells treated with nucleolus bright green (NBG) and TEG$_8$-N14 after subtracting the background. All cells were treated with a fixative and imaged using volumetric mode. Scale bar: 10 μm.

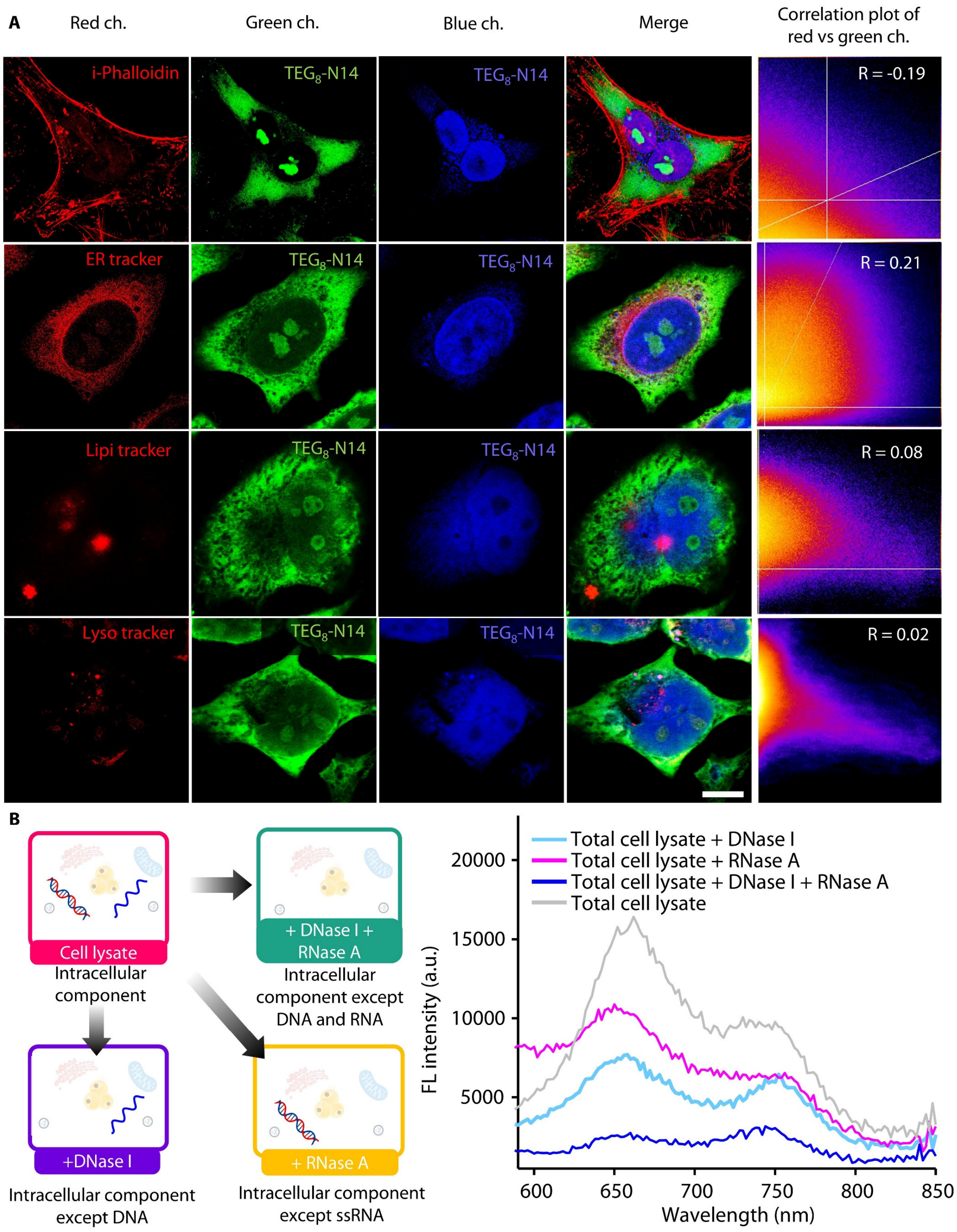

Fig. S9. Specificity of TEG8-N14 for binding to nucleic acids over other biomolecules. (**A**) Co-staining of organelles. Red: i-Phalloidin (λ_{ex}: 405 nm, λ_{em}: 415 – 630 nm), ER Tracker (λ_{ex}: 405 nm, λ_{em}: 415 – 630 nm), Lipi Tracker (λ_{ex}: 450 nm, λ_{em}: 500 – 600 nm), or Lyso Tracker (λ_{ex}: 450 nm, λ_{em}: 500 – 600 nm); green: TEG8-N14 (λ_{ex}: 640 nm, λ_{em}: 650 – 720 nm); and blue: TEG8-N14 (λ_{ex}: 730 nm, λ_{em}: 740 – 850 nm). (**B**) Emission spectra of HeLa cell lysate without and with nuclease treatment, followed by addition to TEG8-N14.

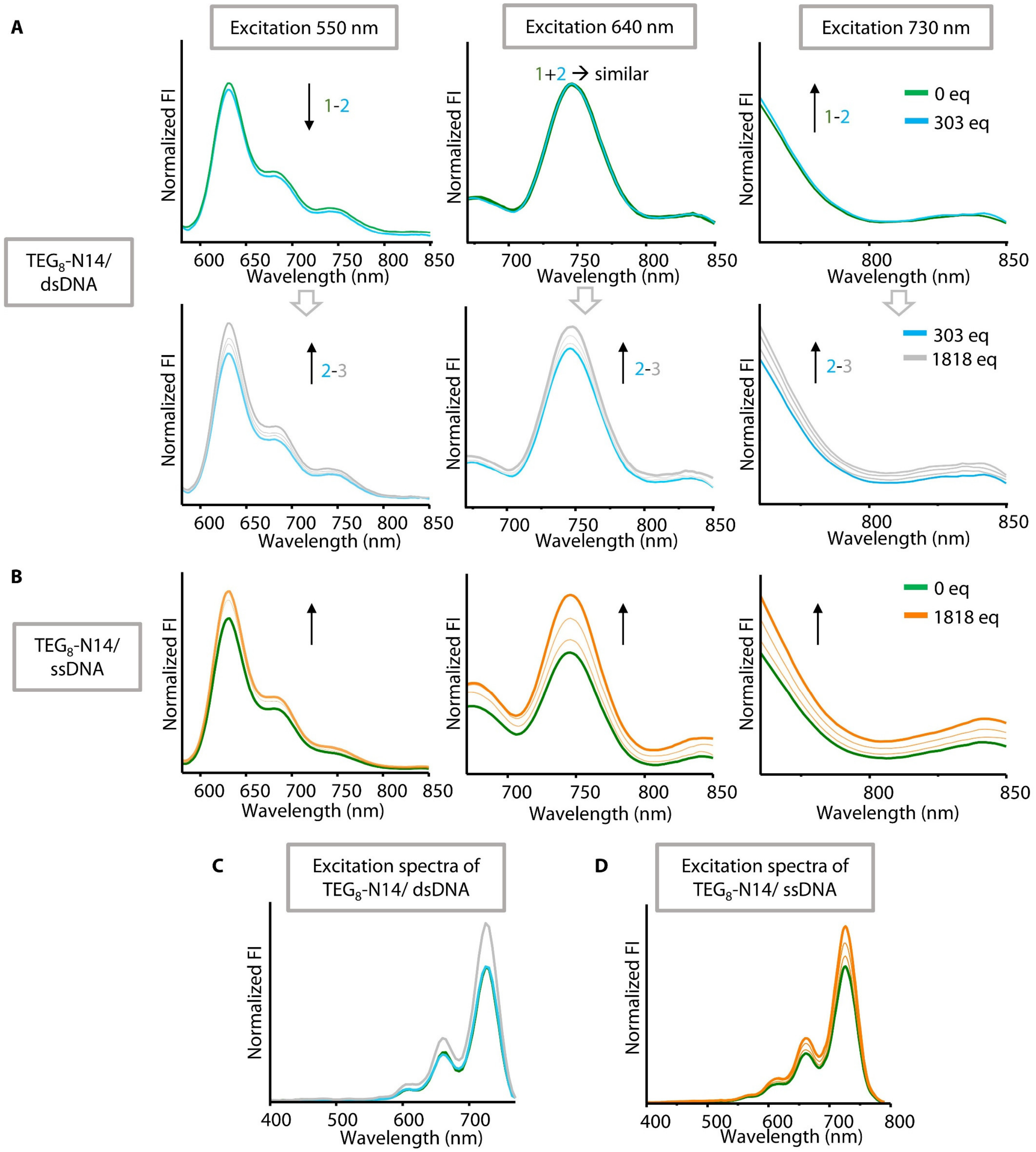

Fig. S10. Fluorescence spectra of TEG$_8$-N14 and stepwise addition of commercialized dsDNA and ssDNA in Tris-EDTA buffer solution (pH 8.0). Fluorescence emission spectra of TEG$_8$-N14 with stepwise addition of dsDNA (**A**) and ssDNA (**B**) with different excitation wavelength. Fluorescence excitation spectra (λ_{em} = 820 nm) of TEG$_8$-N14 after mixing with dsDNA (**C**) and ssDNA (**D**). The equivalent is calculated using a ratio of base pairs of nucleic acid to one dye molecule.

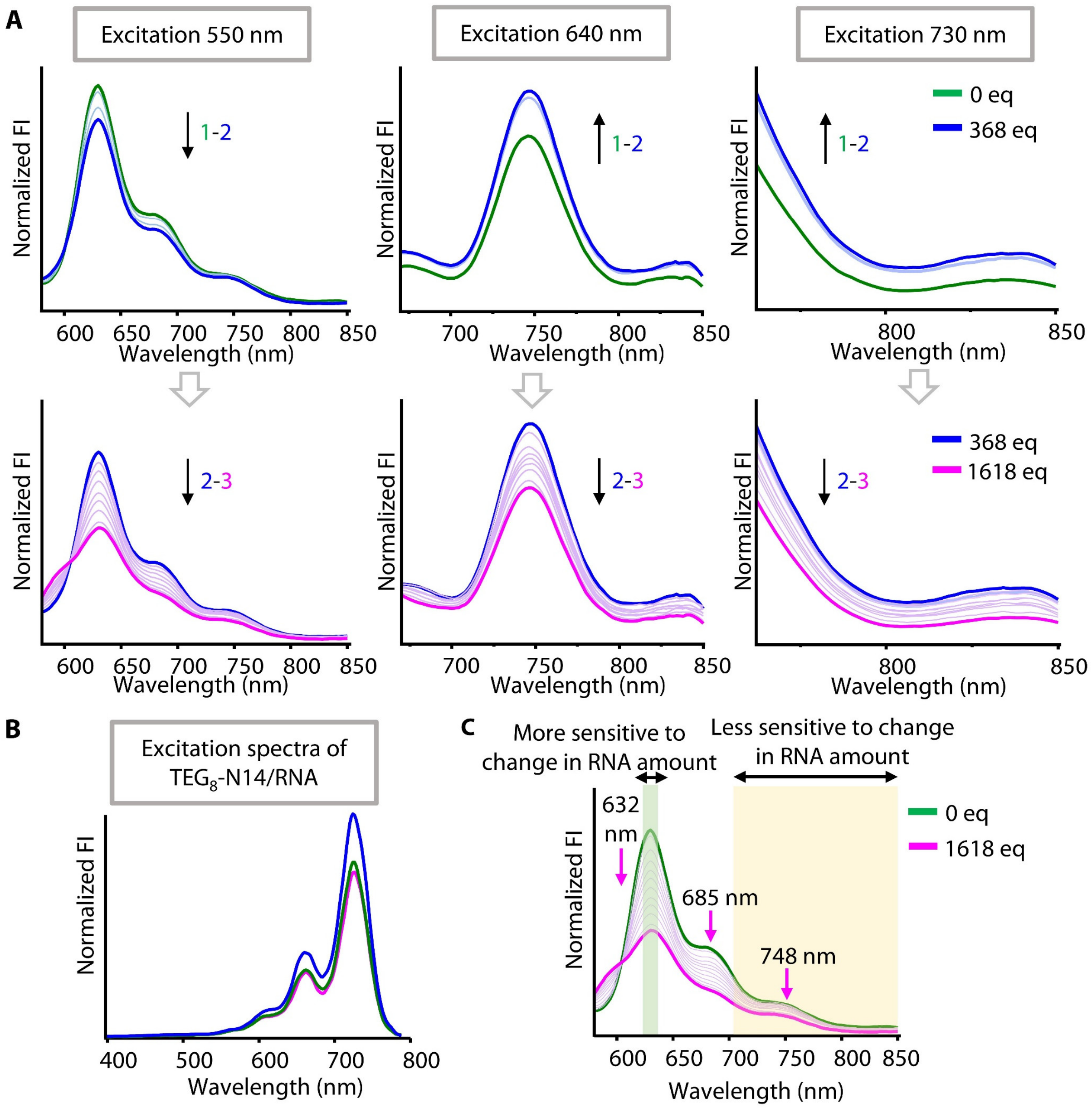

Fig. S11. Fluorescence spectra of TEG$_8$-N14 and its derivatives and commercialized RNA in Tris-EDTA buffer solution (pH 8.0). (**A**) Fluorescence emission spectra after titration of TEG$_8$-N14 with stepwise addition of RNA. (**B**) Fluorescence excitation spectra of TEG$_8$-N14 with stepwise addition of RNA. (**C**) Summary of fluorescence emission after titration with RNA under excitation of 550 nm. The equivalent is calculated using a ratio of base pairs of nucleic acid to one dye molecule.

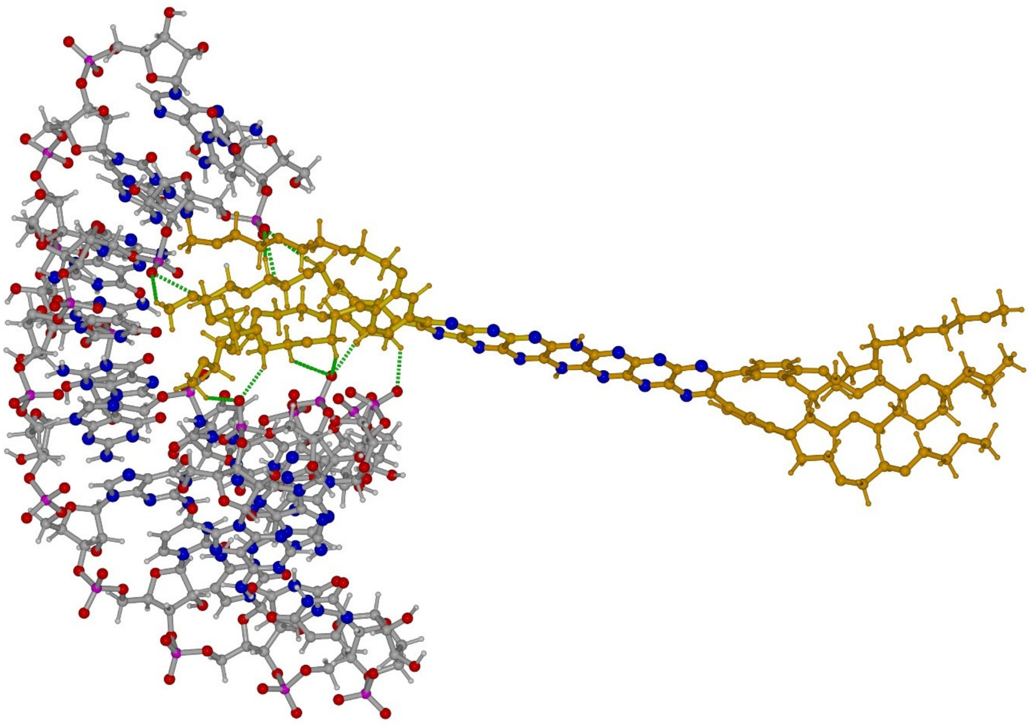

Fig. S12. **Interactions between dsRNA and TEG$_8$-N14 in implicit water environment.** Notably, main interactions are C-H…O hydrogen bonds between oligoethylene glycol chains and the phosphate backbones with N14 deflected away from the nucleic acid. Green broken bonds indicate C-H…O hydrogen bonds.

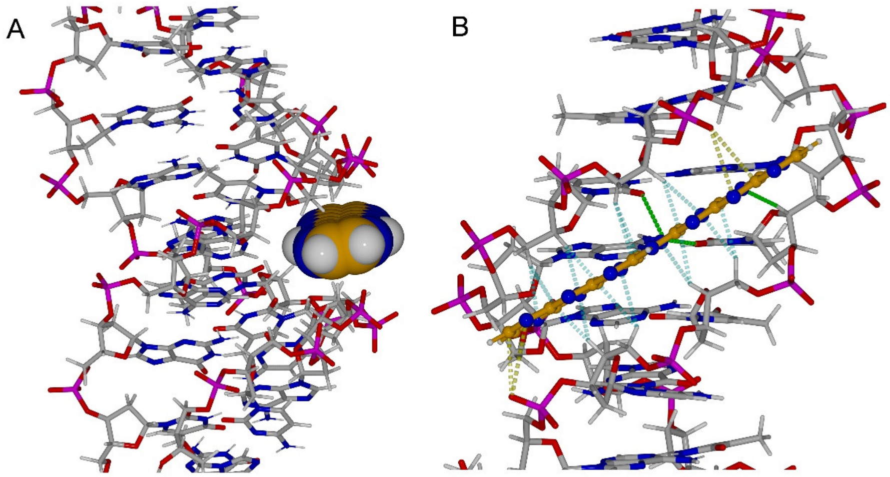

Fig. S13. **Additional representations of the minor groove binding of TEG$_8$-N14 to DNA.** (**A**) Mixed representation with N14 depicted in space-filling mode illustrating the effective fit of N14 in the minor groove. (**B**) N14 core-minor groove interactions: green broken bonds: N14 N-H…O hydrogen bonds; blue broken bonds: C-H…N hydrogen bonds; yellow broken bonds: P-O…π (anion-π) interactions.

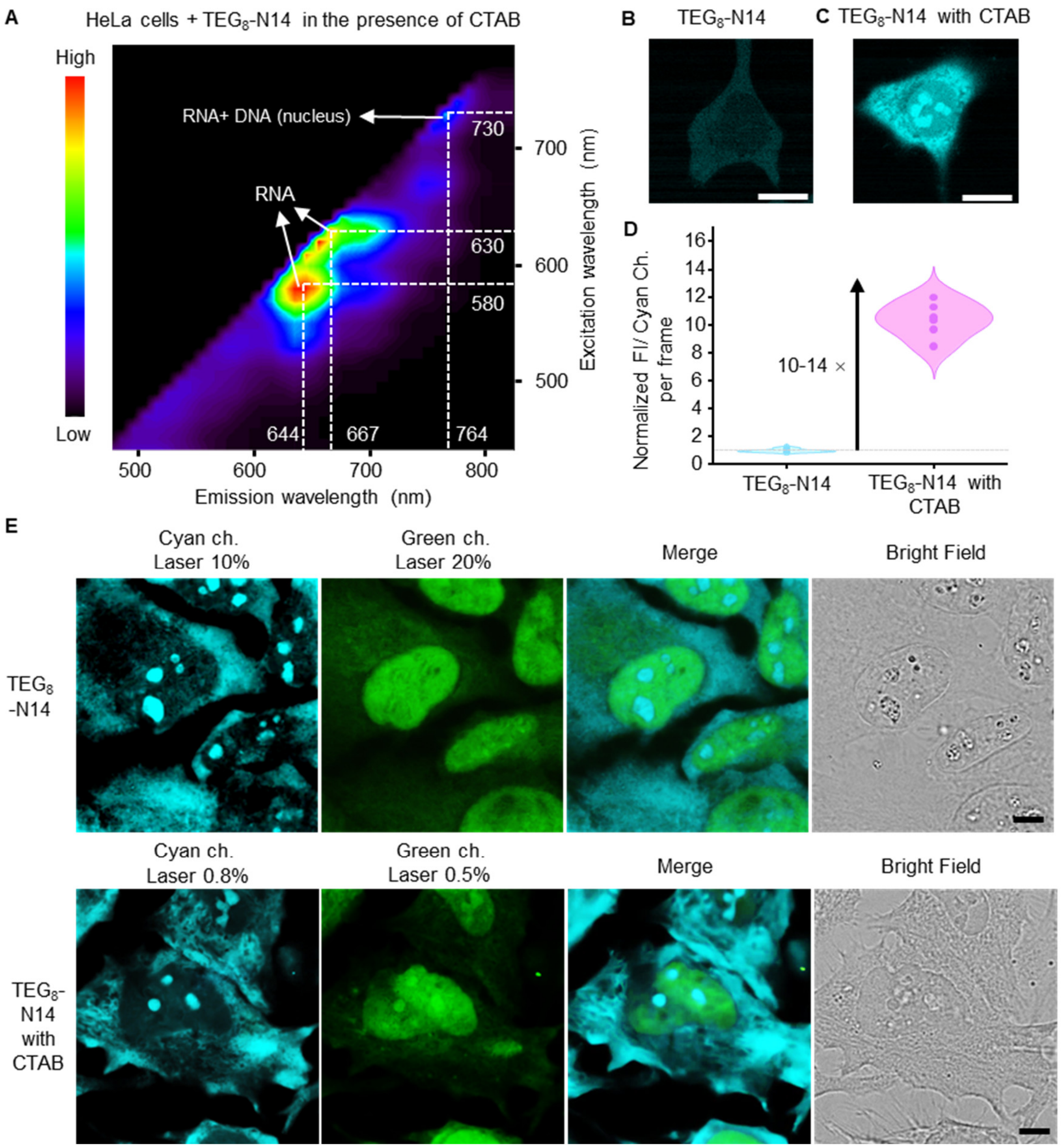

Fig. S14. Brightness comparison of TEG8-N14 with and without CTAB in fixed HeLa cells. **(A)** Representative two-dimensional fluorescence images of HeLa cells stained with TEG8-N14 in the presence of CTAB. **(B)** Representative confocal fluorescence images of HeLa cells stained with TEG8-N14 (**A**) and in the presence of CTAB (**B**) taken with identical microscope settings (λ_{ex} = 640 nm; λ_{em} = 650 – 720 nm) are shown. (**C**) Brightness of the sum projection images within the cells is normalized. Data are presented as mean ± S.D. (n > 150 cells across five different fields of view). Each dot represents one field of view. Scale bar, 10 µm. (**D**) Compatibility of TEG8-N14 with and without CTAB was observed using a different microscope (Olympus FV3000). Cyan channel (λ_{ex} = 640 nm and λ_{em} = 650 – 720 nm) and green channel (λ_{ex} = 730 nm and λ_{em} = 740 – 900 nm). Scale bar, 5 µm. All cells were treated with a fixative and imaged using volumetric mode.

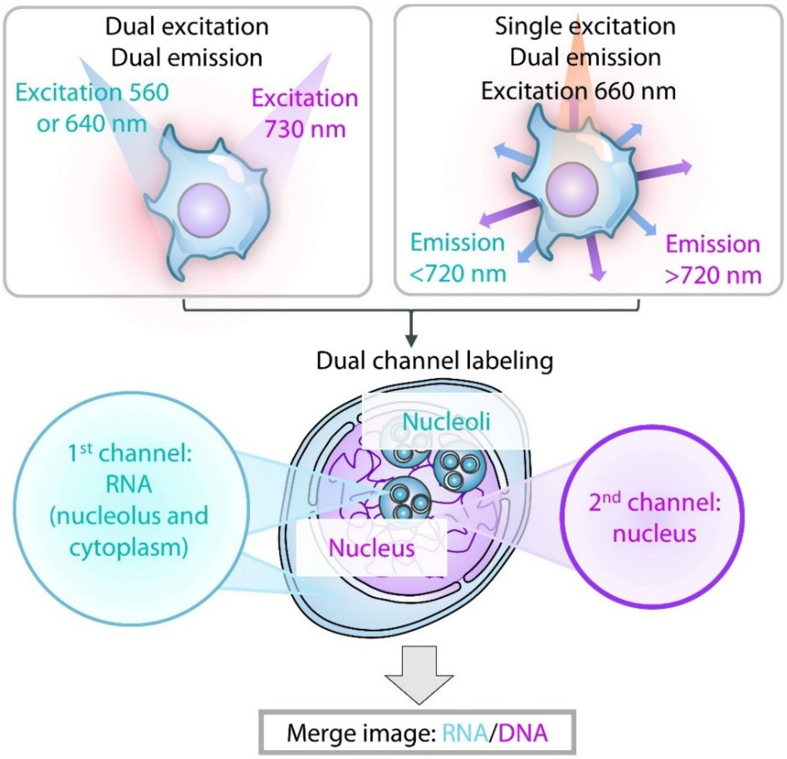

Fig. S15. Schematic illustration of optional spectrum to detect different organelles in cells stained with TEG_8-N14.

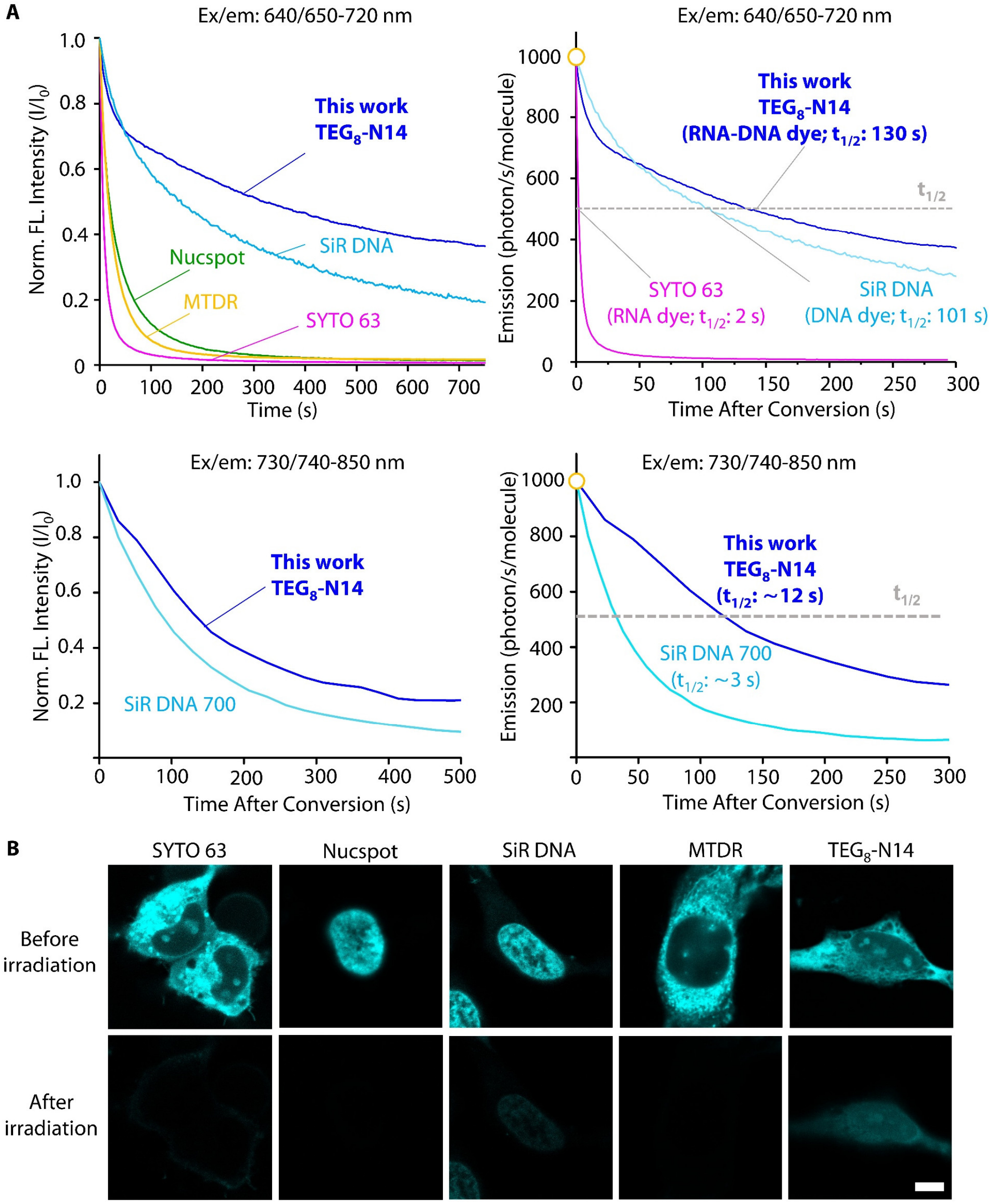

Fig. S16. Comparison of photobleaching curves. (**A**) Fixed HeLa cells were labelled and imaged continuously with an illumination intensity of ~3.3 or ~26 W cm^{-2}. Photostability data were presented as normalized values (left), and converted (right) values. The data shown at the top were collected using the cyan channel (λ_{ex} = 640 nm, λ_{em} = 650–670 nm), while the data at the bottom awas collected using the magenta channel (λ_{ex} = 730 nm, λ_{em} = 740–850 nm). The data are shown as intensity relative to the normalized total exposure time, assuming an initial emission rate of 1,000 photons per second per molecule. (**B**) Representative confocal images of HeLa cells before and after continuous irradiation. Scale bar: 10 µm. Note: For this photostability assay, TEG_8-N14 was added to the CTAB solution to enhance staining efficiency, making it comparable to other commercial dyes. Mitotracker Deep Red, MTDR.

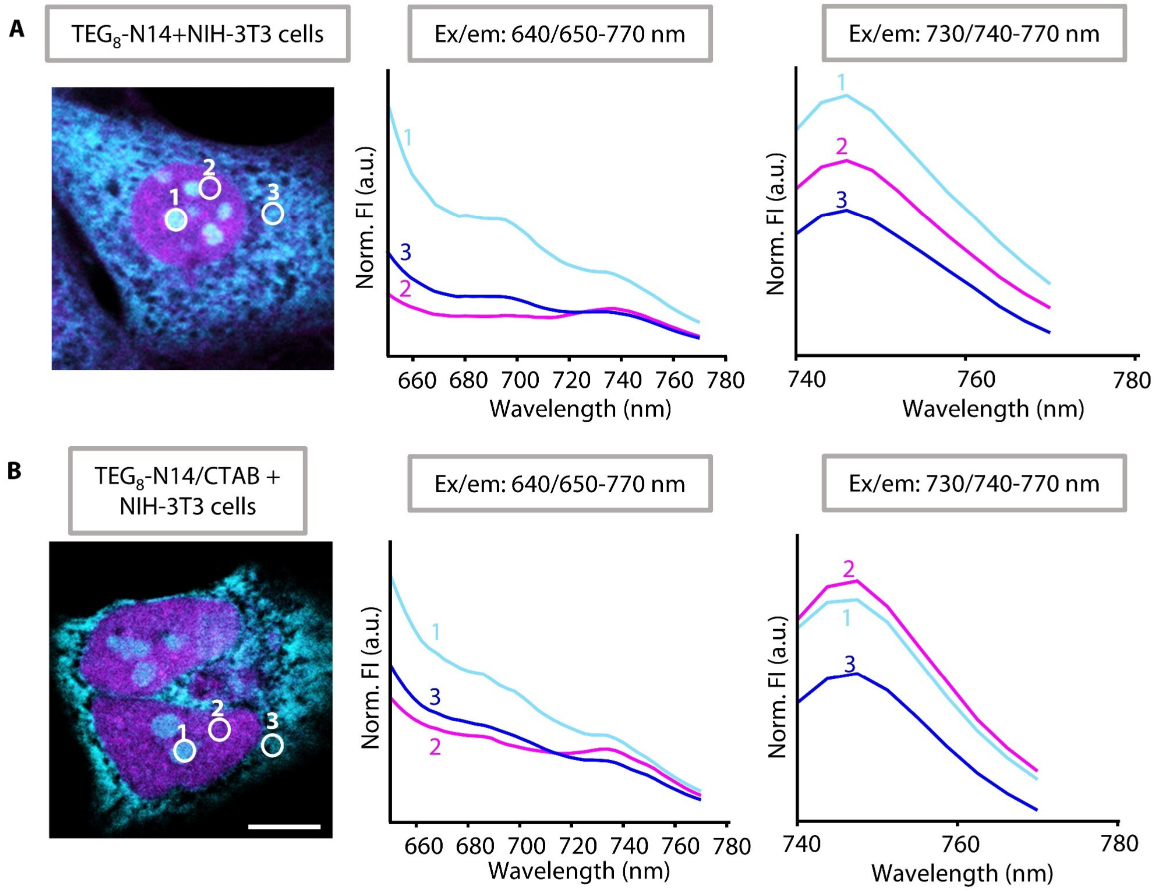

Fig. S17. Fluorescence emission spectra of fixed NIH-3T3 cells treated with TEG_8-N14 with and without addition of CTAB. Unmixed image of NIH-3T3 cells (left) using the reference spectra of selected pixels in areas 1 (nucleolus), 2 (nucleus), and 3 (cytoplasm). The cells were treated with TEG_8-N14 without CTAB (**A**) and with addition of CTAB (**B**) after fixation of NIH-3T3 cells. The emission spectra for the respective areas, with different excitation wavelengths, are displayed on the right. The similar results were obtained from two independent experiments. Scale bar: 10 µm. All cells were treated with a fixative and imaged using volumetric mode.

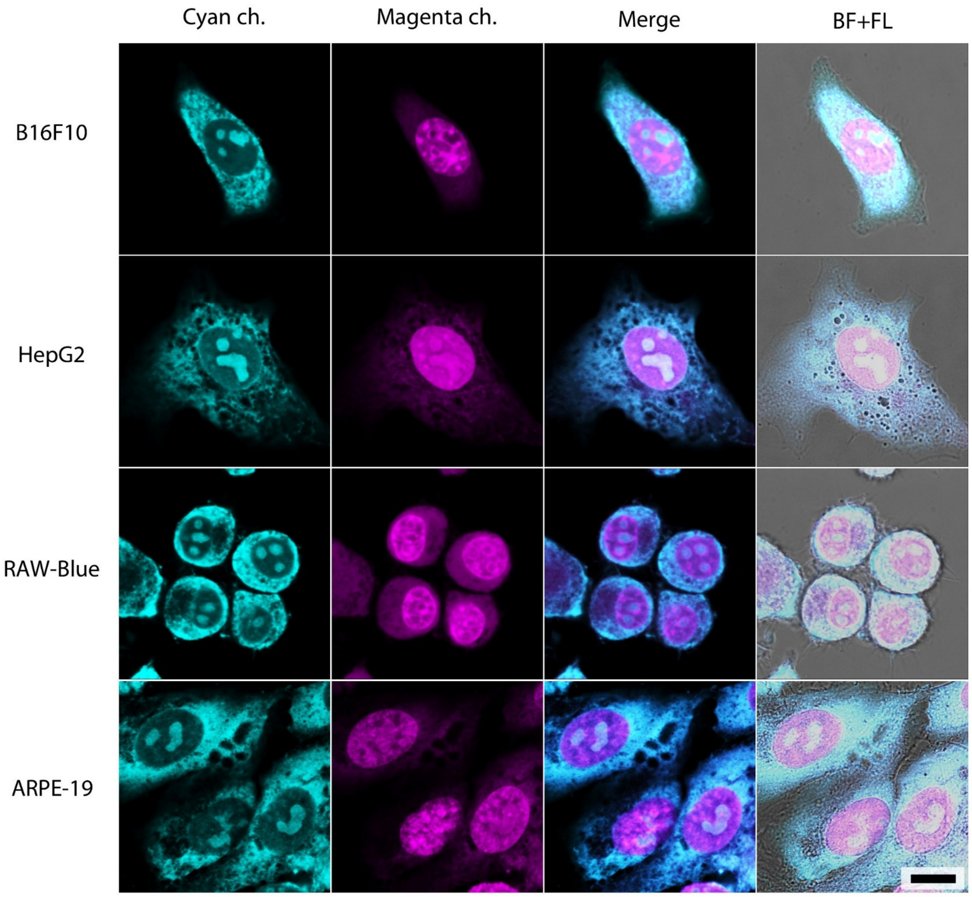

Fig. S18. Co-localization imaging of various cell types based on morphology or source after staining with TEG$_8$-N14. From top to bottom: B16F10, HepG2, RAW Blue, ARPE-19 cells. From left to right: cyan channel (λ_{ex} = 640 nm; λ_{em} = 650 – 720 nm), magenta channel (λ_{ex} = 730 nm; λ_{em} = 740 – 850 nm), merge, and bright field. BF: bright-field, FL: Fluorescence. Scale bar: 10 µm. All cells were treated with a fixative and imaged using volumetric mode.

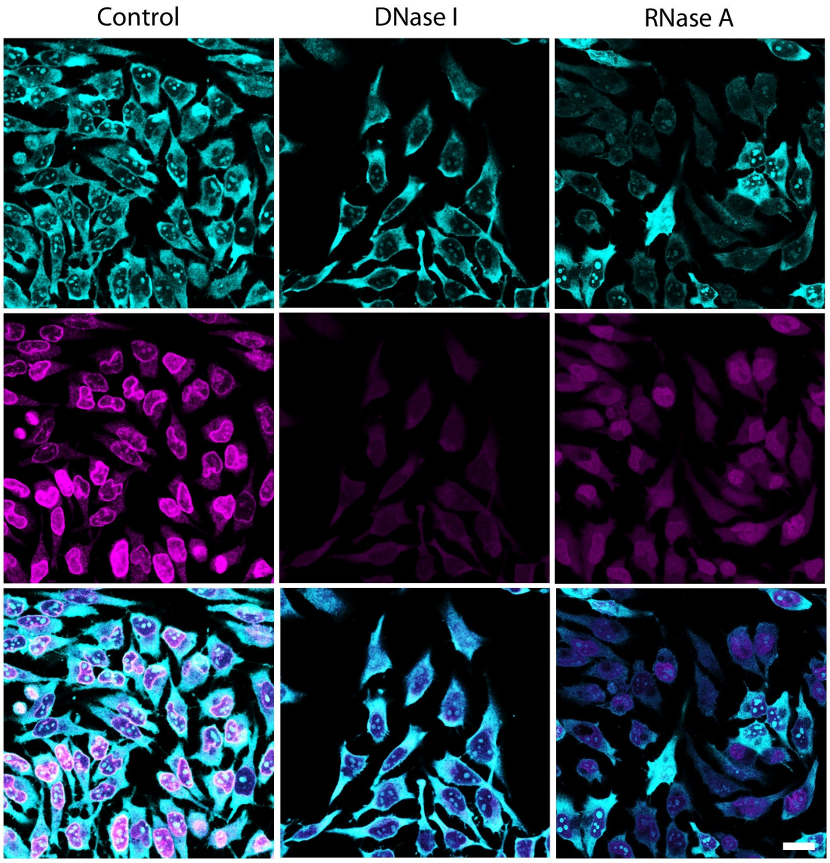

Fig. S19. DNA-RNA sensitivity properties of TEG$_8$-N14. Representative confocal images of HeLa cells untreated (left), DNase I-treated (middle) and RNase A-treated (right), followed by staining with TEG$_8$-N14, followed by imaging in the cyan channel (λ_{ex} = 640 nm; λ_{em} = 650 – 720 nm) and magenta channel (λ_{ex} = 730 nm, λ_{em} = 740 – 850 nm). Scale bar: 10 µm. All cells were treated with a fixative and imaged using volumetric mode.

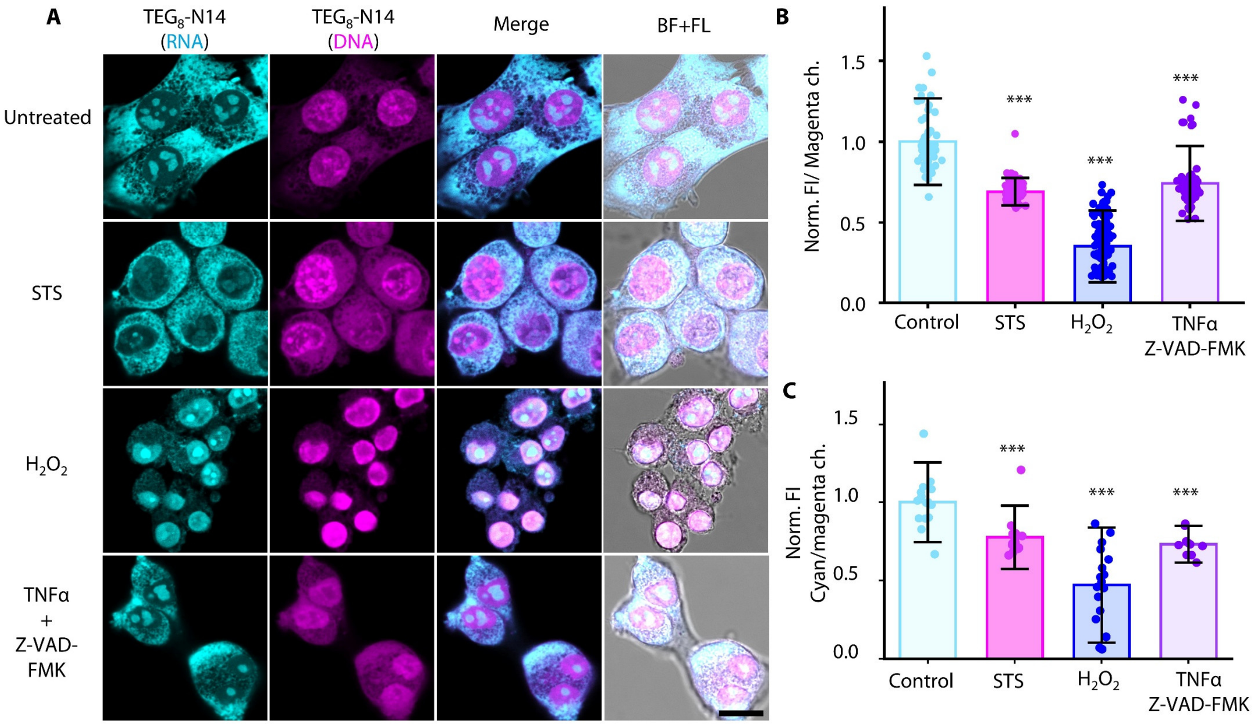

Fig. S20. Fluorescence emission spectra of fixed NIH-3T3 cells undergo apoptosis, necrosis, and necroptosis. (**A**) Representative confocal images of NIH-3T3 cells after 6 hours of incubation with STS, H_2O_2, and TNF-α + Z-VAD-FMK. Scale bar, 10 µm. STS, staurosporine. BF: bright-field, FL: Fluorescence. Intracellular signal intensities quantification in the magenta (**B**) and cyan-to-magenta (**C**) channels after NIH-3T3 cells were injured for 6 hours. The intensities are normalized in each imaging channel to the average of the control group. Data are means ± S.D. of three independent experiments (n > 100 cells per experiment). Significance levels are indicated as $**p < 0.01$ and $***p < 0.001$, determined using an unpaired two-tailed Student's t-test. STS, staurosporine. FL, fluorescence. a.u., arbitrary units. All cells were treated with a fixative and imaged using volumetric mode.

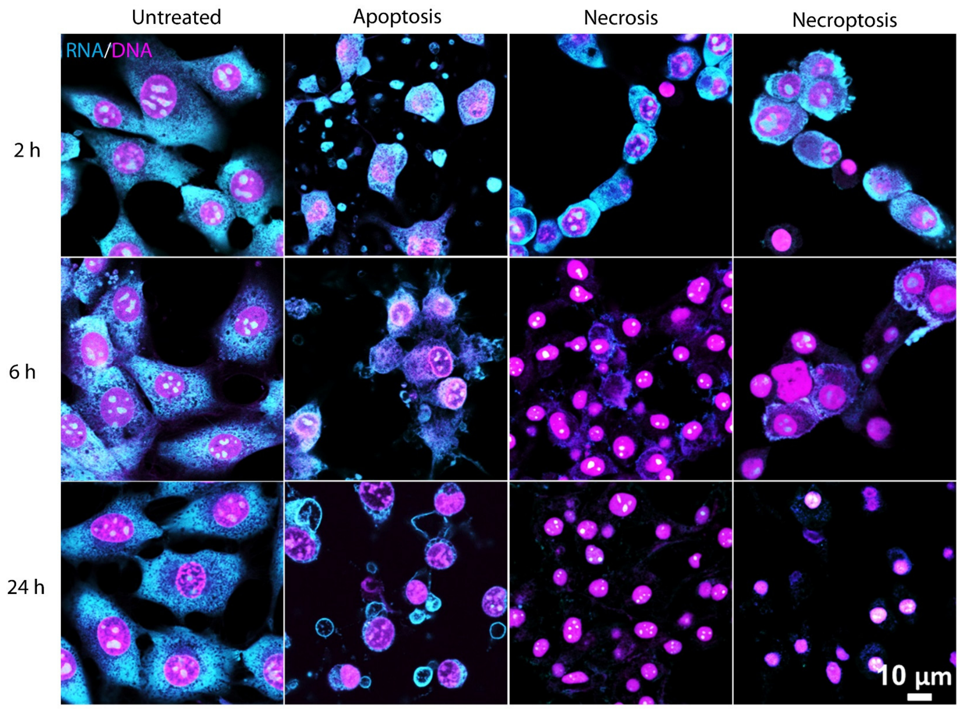

Fig. S21. TEG$_8$-N14 enables labeling of different states of NIH-3T3 cells after the induction of apoptosis, necrosis, or necroptosis. Representative confocal images of NIH-3T3 cells without and with treatment of STS, H_2O_2, and TNF-α+Z-VAD-FMK to induce apoptosis, necrosis, and necroptosis, respectively. The images were prepared by merging the cyan (λ_{ex} = 640 nm; λ_{em} = 650 – 720 nm) and magenta (λ_{ex} = 730 nm; λ_{em} = 740 – 850 nm) channels. Scale bar: 10 µm. STS, staurosporine. All cells were treated with a fixative and imaged using volumetric mode.

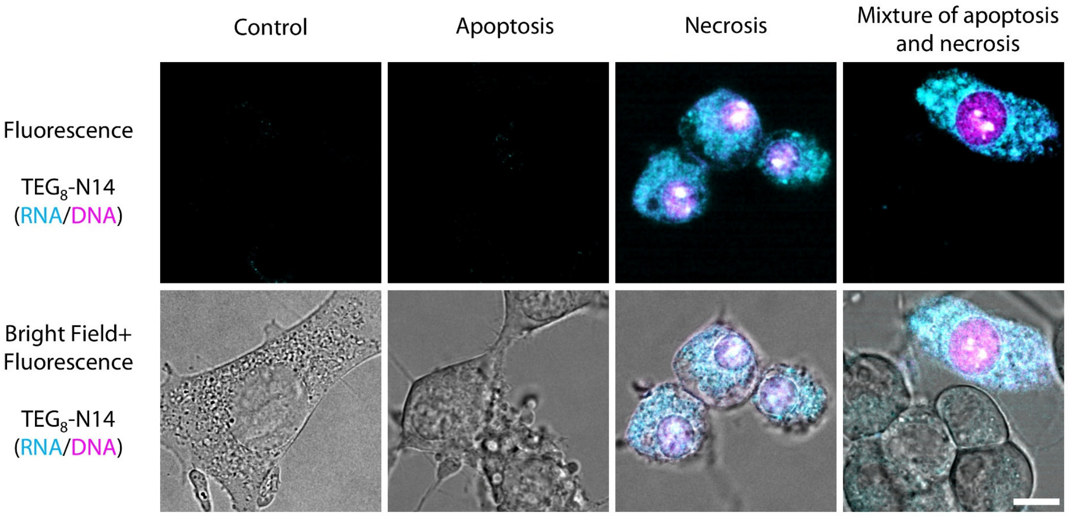

Fig. S22. TEG8-N14 as selective necrotic labeling dyes. Representative confocal images of NIH-3T3 cells without and with treatment of STS, H_2O_2, and TNF-α+Z-VAD-FMK to induce apoptosis, necrosis, and necroptosis, respectively. The images were prepared by merging the cyan (λ_{ex} = 640 nm; λ_{em} = 650 – 720 nm) and magenta (λ_{ex} = 730 nm; λ_{em} = 740 – 850 nm) channels. Scale bar: 10 µm. STS, staurosporine. All cells were directly stained without any fixative and imaged using volumetric mode.

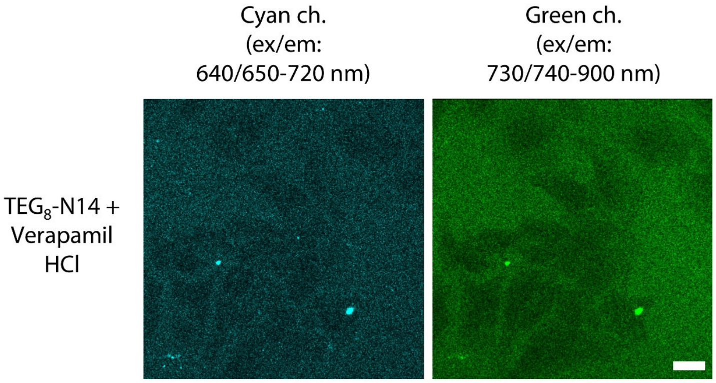

Fig. S23. Representative confocal images of live HeLa cells stained with a mixture of TEG8-N14 and an efflux pump inhibitor. Observations were performed in two channels: cyan (λ_{ex} = 640 nm; λ_{em} = 650 – 720 nm) and magenta (λ_{ex} = 730 nm; λ_{em} = 740 – 900 nm). Scale bar: 10 µm. All cells were directly stained without any fixative and imaged using volumetric mode.

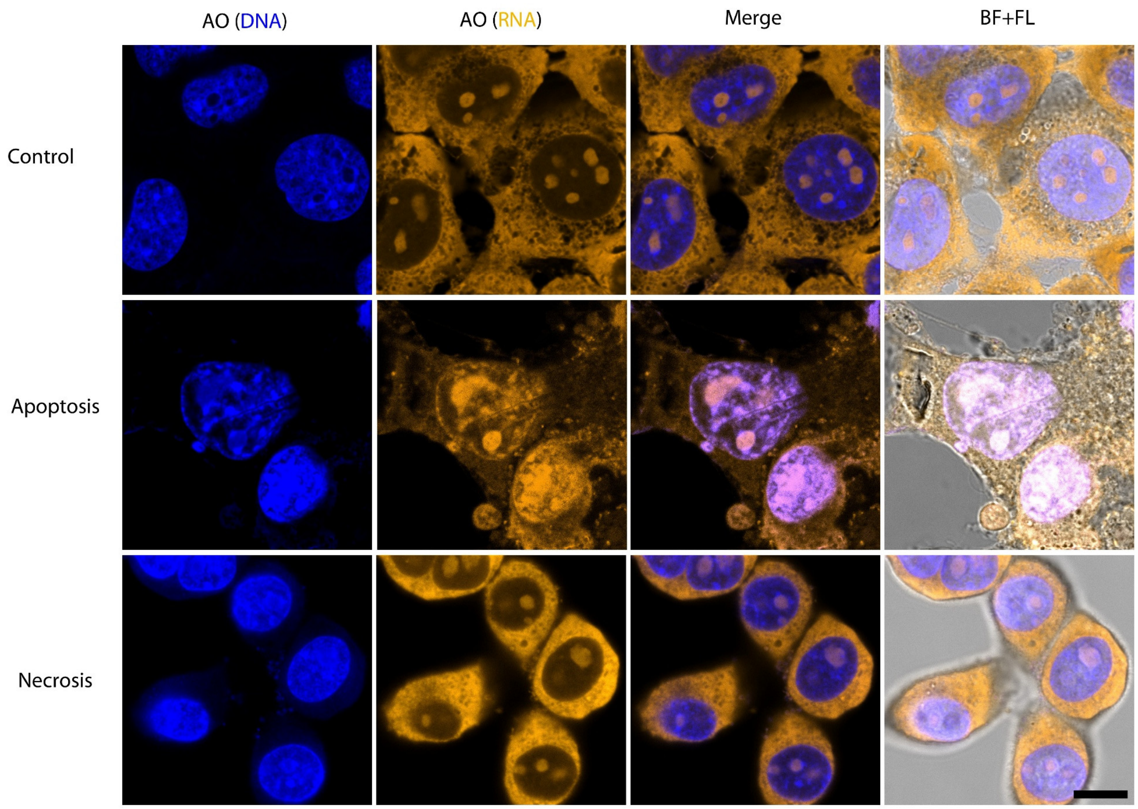

Fig. S24. Confocal images of different states of NIH-3T3 cells after the induction of apoptosis, necrosis, or necroptosis labeled by AO. Images were prepared by merging the blue (λ_{ex} = 457 nm; λ_{em} = 467 – 550 nm) and yellow (λ_{ex} = 457 nm; λ_{em} = 600 – 750 nm) channels. Scale bar: 10 µm. All cells were directly stained without any fixative and imaged using volumetric mode.

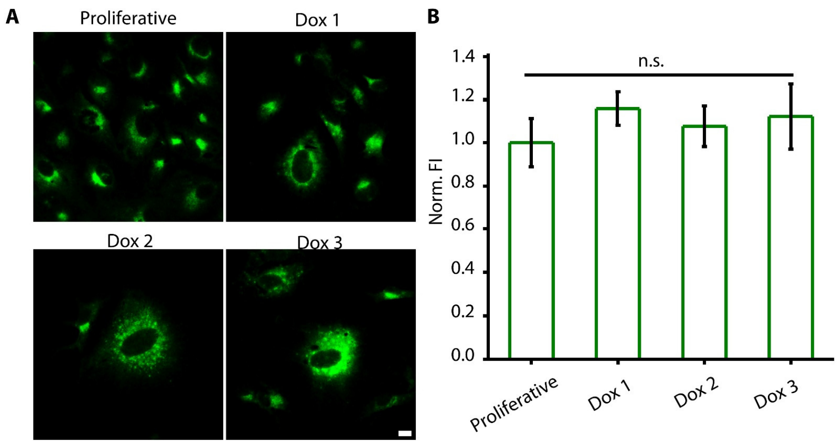

Fig. S25. Beta-galactosidase staining in proliferative and senescent in ARPE-19 cells. (A) Representative confocal images of ARPE-19 cells with and without Dox induction. The observations were made at λ_{ex} = 488 nm; λ_{em} = 500 – 550 nm. Scale bar: 10 µm. (**B**) Quantitative analysis of changes in proliferative and senescent cells measured using a microplate reader. n.s.: notable signal. All cells were treated with a fixative and imaged using volumetric mode.

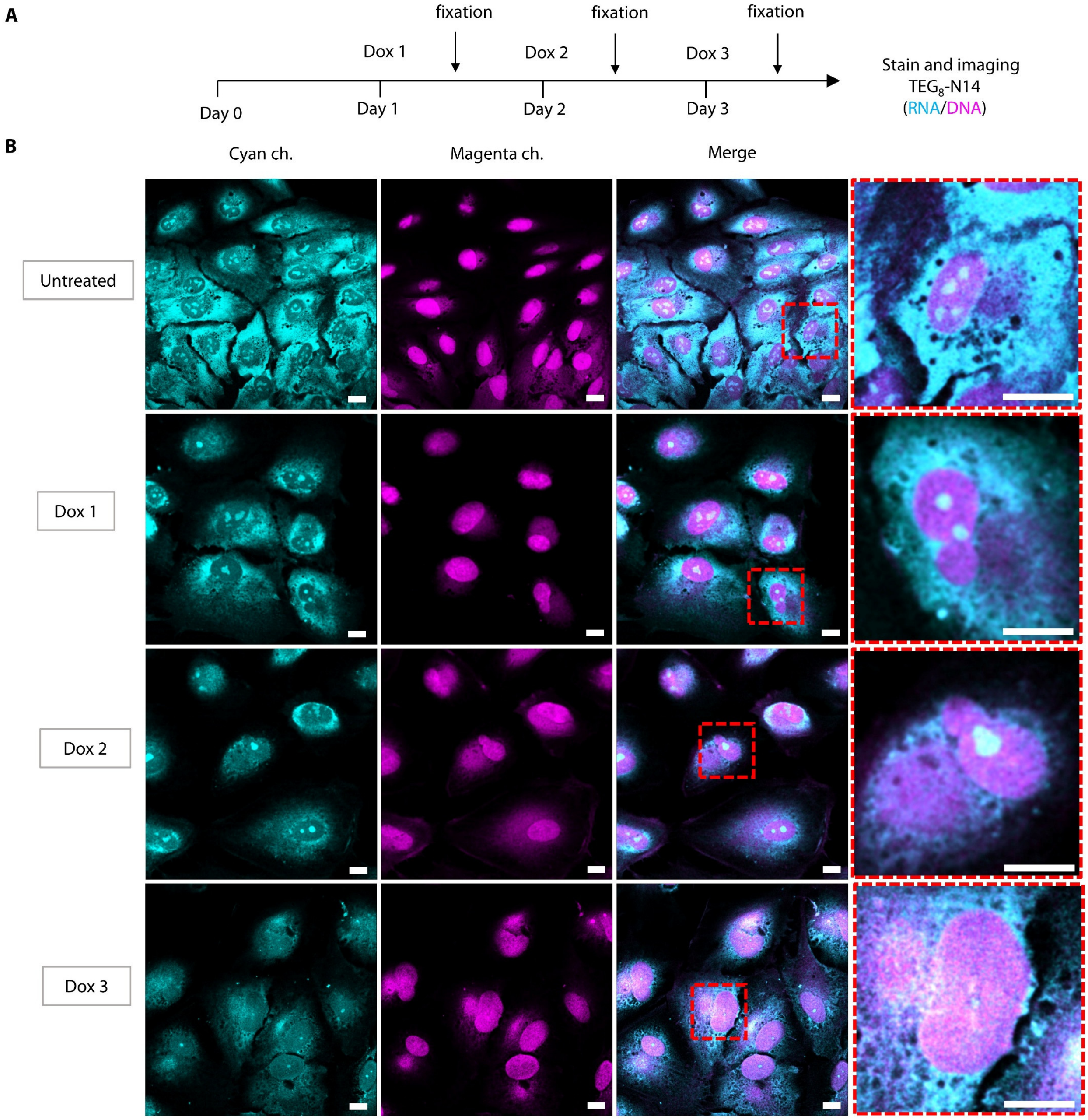

Fig. S26. Confocal images of ARPE-19 cells after incubation with doxorubicin for three consecutive days. (**A**) Schematic representation of the chemically induced senescence process and the imaging workflow after staining with TEG8-N14. (**B**) From left to right: cyan channel (λ_{ex} = 640 nm; λ_{em} = 650 – 720 nm), magenta channel (λ_{ex} = 730 nm = λ_{em} = 740 – 850 nm), channel merged image, and high magnification image of merged channel. Scale bar, 10 µm. All cells were treated with a fixative and imaged using volumetric mode.

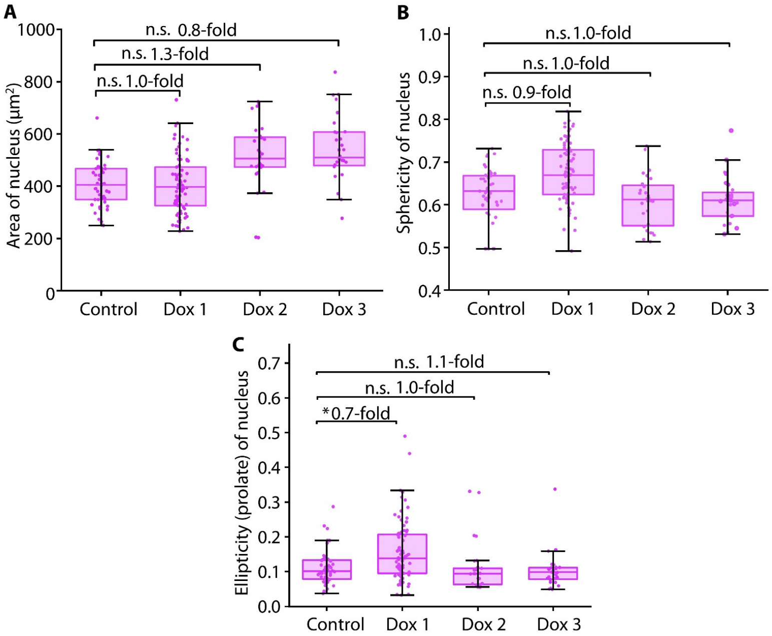

Fig. S27. Quantitative analysis of changes in senescent cells. Analysis of change in nucleus area (**A**), sphericity (**B**), and ellipticity (**C**). Changes are displayed as mean ± S.D. and plotted from three independent experiments, based on data from more than 30 cells per condition. Significance levels are indicated as *p < 0.05, **p < 0.01, ***p < 0.001, determined using an unpaired two-tailed Student's t-test, n.s.: notable signal.

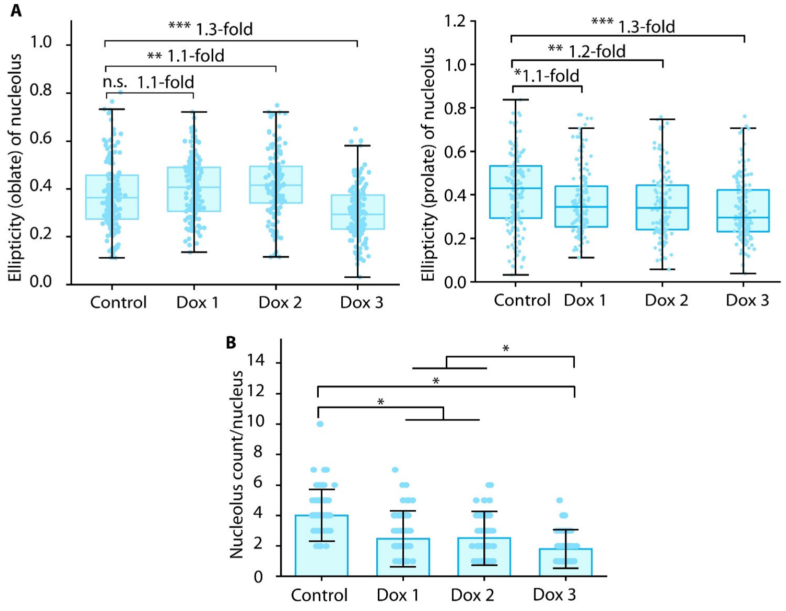

Fig. S28. Quantitative analysis of nucleolus changes in senescent cells. Analysis of change in nucleolus number (**A**) and ellipticity (**B**). Changes are displayed as mean ± S.D. and plotted from three independent experiments, based on data from more than 30 cells per condition. Significance levels are indicated as *p < 0.05, **p < 0.01, ***p < 0.001, determined using an unpaired two-tailed Student's t-test, n.s.: notable signal.

Chemical Analytical Data for TEG$_8$-N14

6,7-bis(3,4-bis(2-(2-(2-methoxyethoxy)ethoxy)ethoxy)phenyl)pyrazino[2,3-*b*]pyrazine-2,3-dicarbonitrile

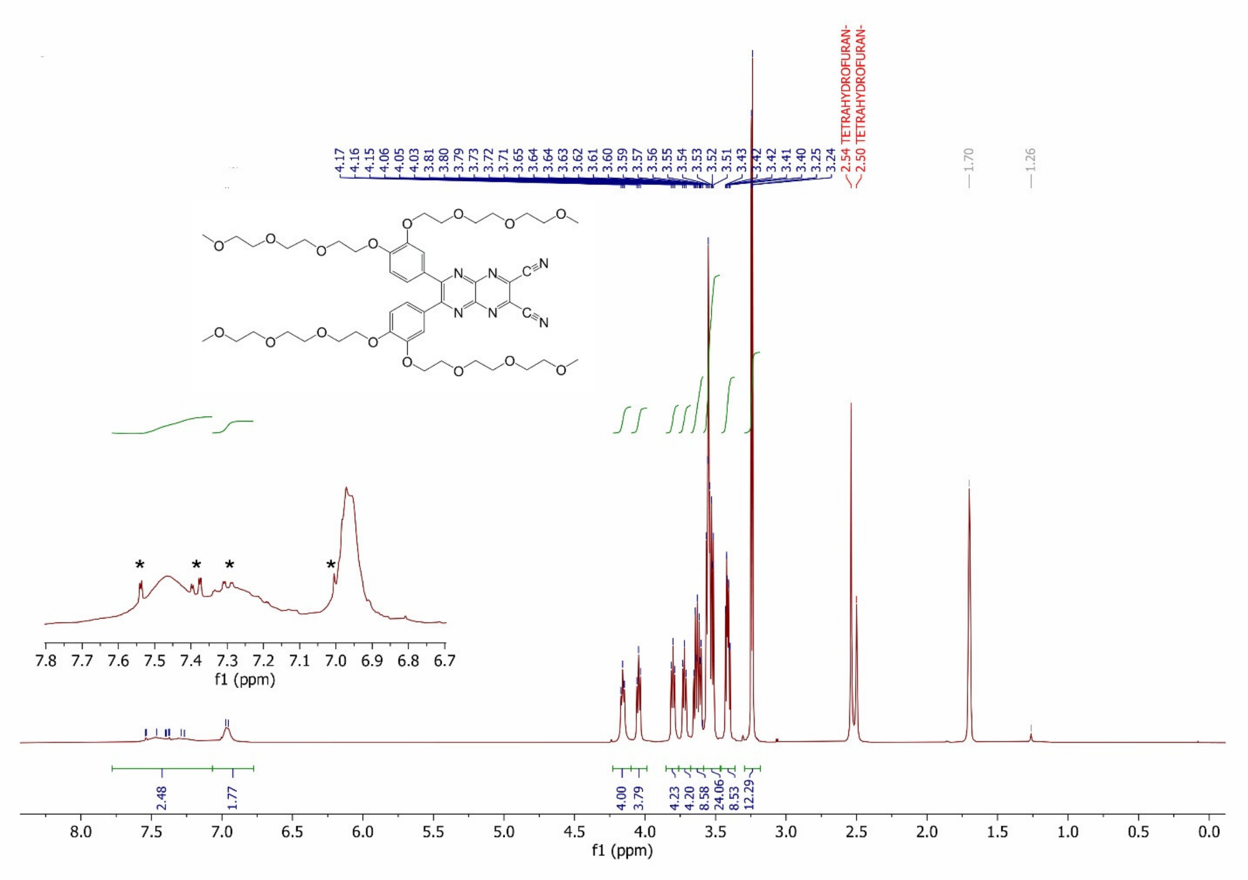

Fig. S29. **^{1}H NMR spectrum of 6,7-bis(3,4-bis(2-(2-(2-methoxyethoxy)ethoxy)ethoxy)phenyl) pyrazino[2,3-*b*]pyrazine-2,3-dicarbonitrile in tetrahydrofuran-*d*$_8$.** Broadening of peaks in the aromatic region is due to aggregation. Asterisks denote the product of the reaction of this dicarbonitrile with methanol yielding 2-methoxy-6,7-bis(3,4-bis(2-(2-(2-methoxyethoxy)ethoxy)ethoxy)phenyl)pyrazino [2,3-*b*]pyrazine-3-carbonitrile (for analyses of this compound prepared in situ in an NMR tube, see Figs. S35-S37).

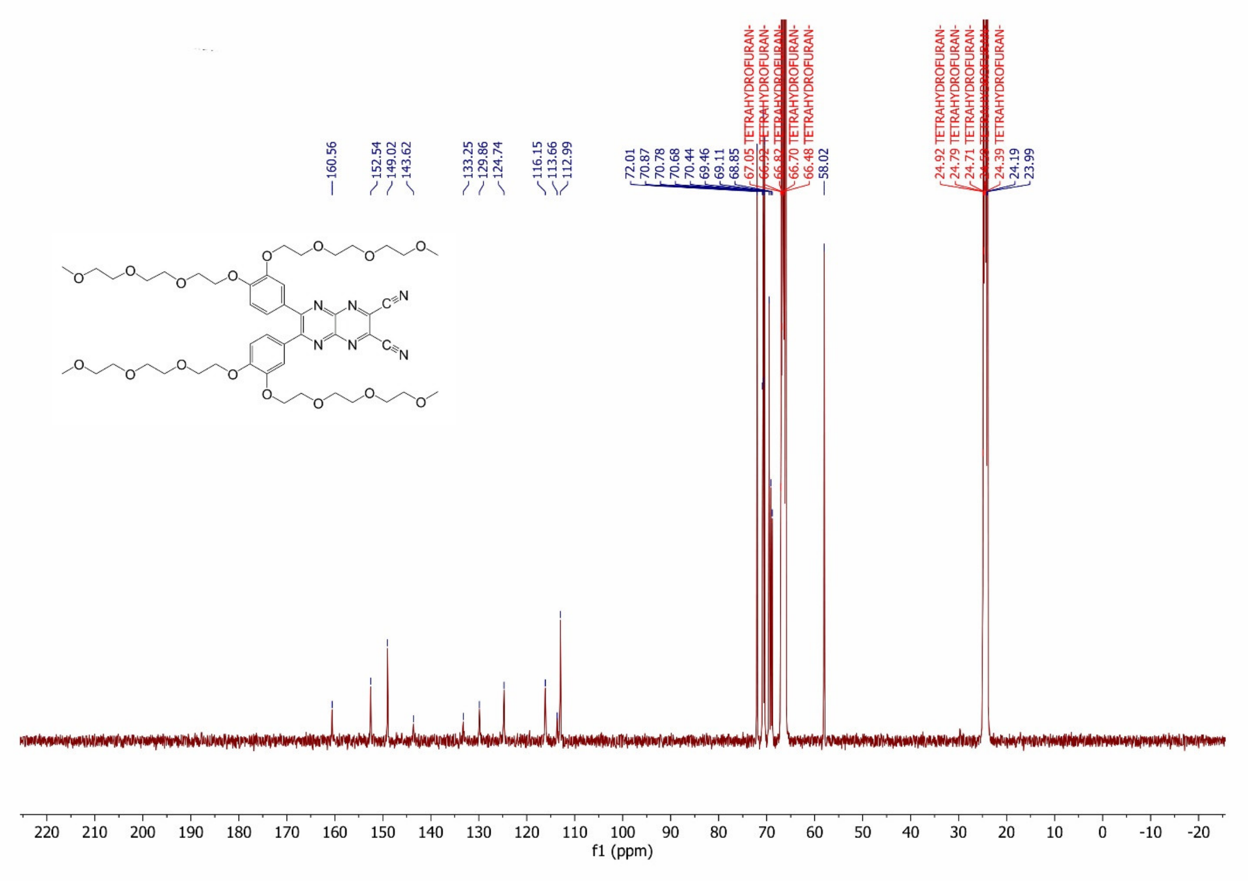

Fig. S30. ^{13}C NMR spectrum of 6,7-bis(3,4-bis(2-(2-(2-methoxyethoxy)ethoxy)ethoxy)phenyl) pyrazino[2,3-*b*]pyrazine-2,3-dicarbonitrile in tetrahydrofuran-d_8.

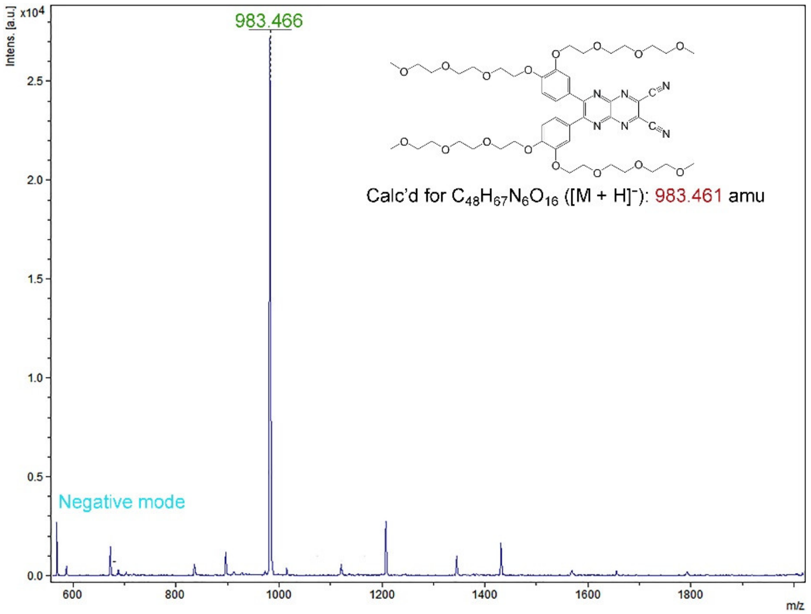

Fig. S31. **MALDI-TOF-MS spectrum (matrix: dithranol; –ve mode) of 6,7-bis(3,4-bis(2-(2-(2-methoxyethoxy)ethoxy)ethoxy) phenyl) pyrazino[2,3-*b*]pyrazine-2,3-dicarbonitrile.**

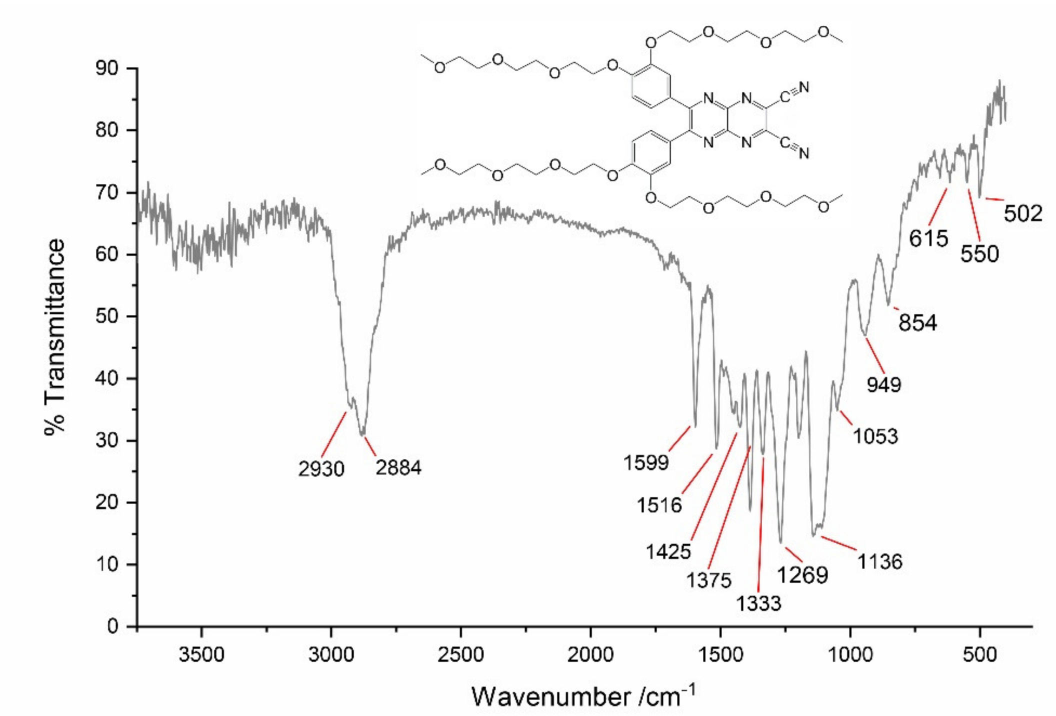

Fig. S32. FTIR spectrum of 6,7-bis(3,4-bis(2-(2-(2-methoxyethoxy)ethoxy)ethoxy)phenyl) pyrazino[2,3-*b*]pyrazine-2,3-dicarbonitrile.

2-Perdeuteriomethoxy-6,7-bis(3,4-bis(2-(2-(2-methoxyethoxy)ethoxy)ethoxy)phenyl)pyrazino[2,3-*b*] pyrazine-3-carbonitrile

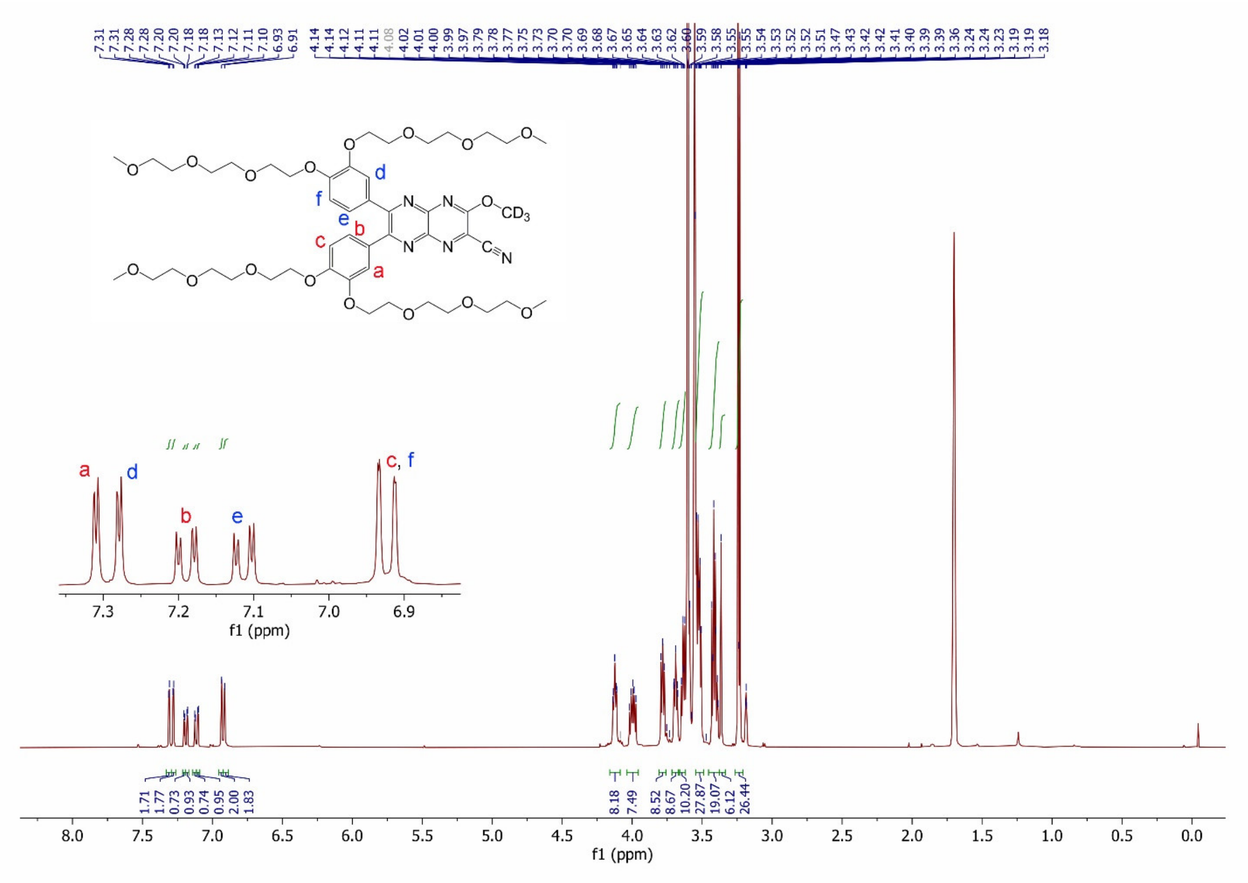

Fig. S33. ^{1}H NMR spectrum of 2-perdeuteriomethoxy-6,7-bis(3,4-bis(2-(2-(2-methoxyethoxy)ethoxy) ethoxy)phenyl)pyrazino[2,3-*b*]pyrazine-3-carbonitrile in tetrahydrofuran-*d*$_8$/CD_3OD with assignments of peaks in the downfield region.

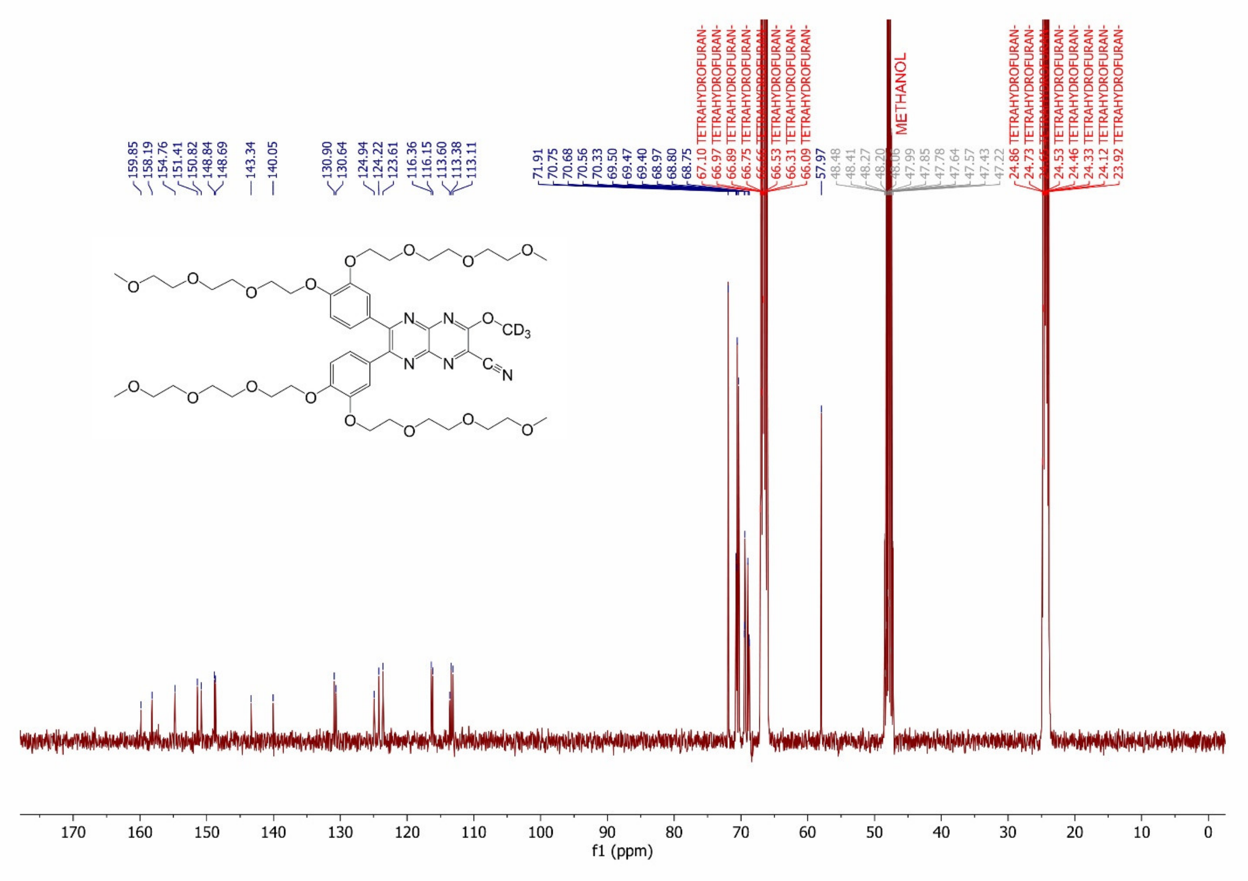

Fig. S34. ^{13}C **NMR spectrum of 2-perdeuteriomethoxy-6,7-bis(3,4-bis(2-(2-(2-methoxyethoxy)ethoxy) ethoxy)phenyl)pyrazino[2,3-*b*]pyrazine-3-carbonitrile in tetrahydrofuran-*d*$_8$/CD_3OD.**

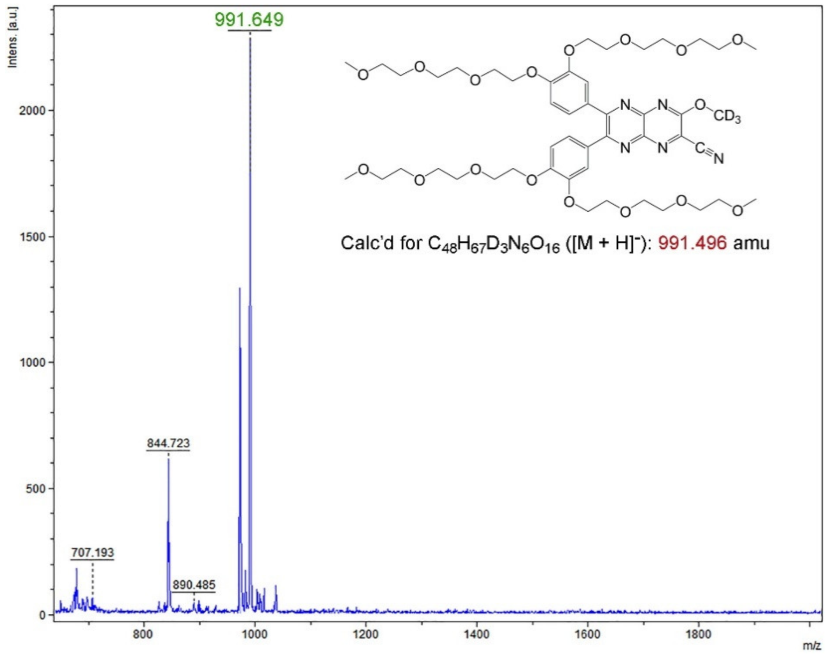

Fig. S35. MALDI-TOF-MS spectrum (matrix: dithranol; –ve mode) of 2-perdeuteriomethoxy-6,7-bis(3,4-bis(2-(2-(2-methoxyethoxy)ethoxy)ethoxy)phenyl)pyrazino[2,3-*b*]pyrazine-3-carbonitrile.

2,3,11,12-Tetrakis(3,4-bis(2-(2-(2-methoxyethoxy)ethoxy)ethoxy)phenyl)-7,16-dihydro-1,4,5,6,7,8,9, 10,13,14,15,16,17,18-tetradecaazaheptacene, TEG$_8$-N14

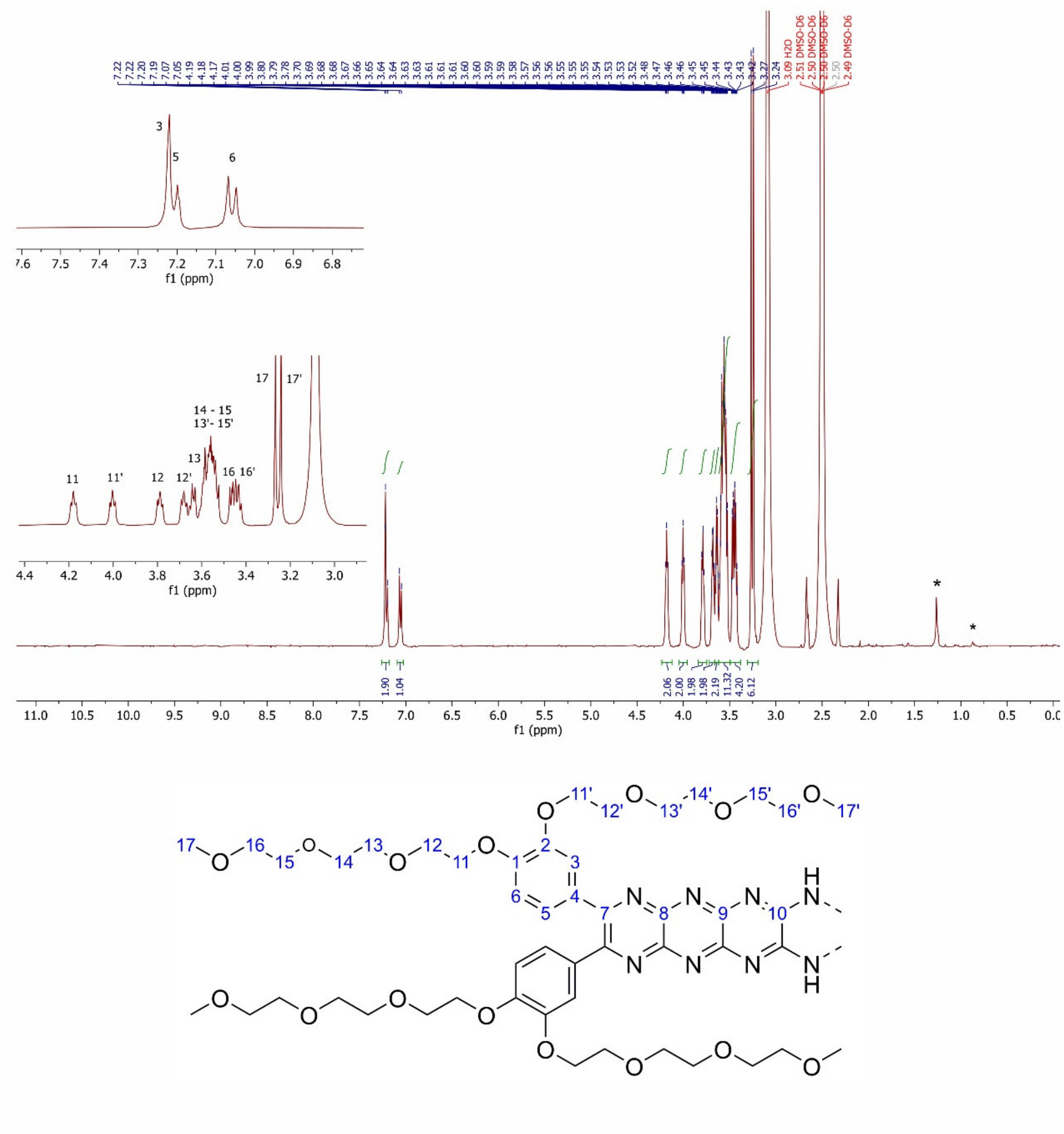

Fig. S36. ^{1}H NMR spectrum of 2,3,11,12-tetrakis(3,4-bis(2-(2-(2-methoxyethoxy)ethoxy)ethoxy)phenyl)-7,16-dihydro-1,4,5,6,7,8,9,10,13,14,15,16,17,18-tetradecaazaheptacene (TEG$_8$-N14) in *d*$_6$-dimethylsulfoxide with complete assignments. Asterisk denotes a non-polar impurity.

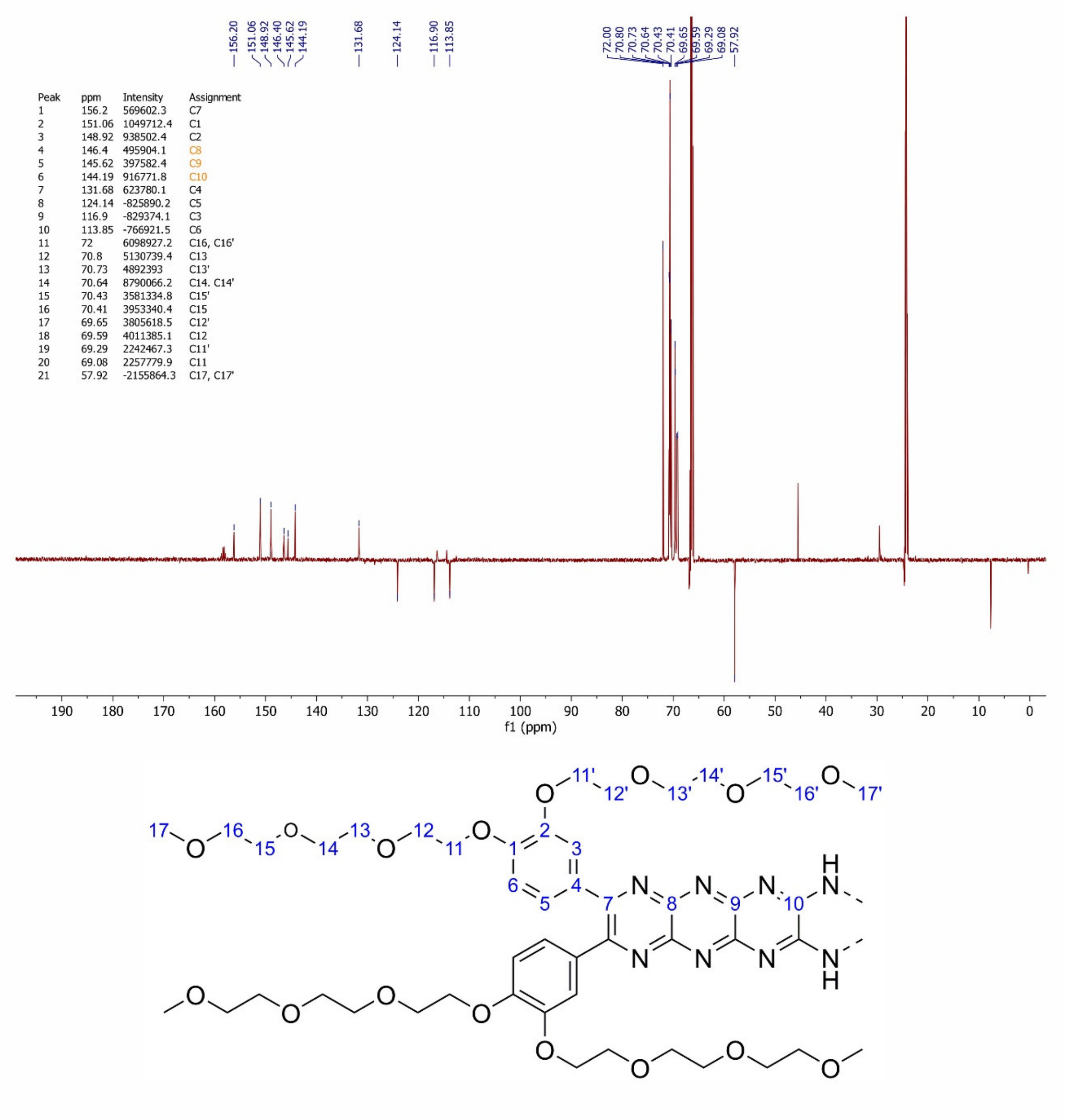

Peak	ppm	Intensity	Assignment
1	156.2	569602.3	C7
2	151.06	1049712.4	C1
3	148.92	938502.4	C2
4	146.4	495904.1	C8
5	145.62	397582.4	C9
6	144.19	916771.8	C10
7	131.68	623780.1	C4
8	124.14	-825890.2	C5
9	116.9	-829374.1	C3
10	113.85	-766921.5	C6
11	72	6098927.2	C16, C16'
12	70.8	5130739.4	C13
13	70.73	4892393	C13'
14	70.64	8790066.2	C14. C14'
15	70.43	3581334.8	C15'
16	70.41	3953340.4	C15
17	69.65	3805618.5	C12'
18	69.59	4011385.1	C12
19	69.29	2242467.3	C11'
20	69.08	2257779.9	C11
21	57.92	-2155864.3	C17, C17'

Fig. S37. ^{13}C APT NMR spectrum of 2,3,11,12-tetrakis(3,4-bis(2-(2-(2-methoxyethoxy)ethoxy)ethoxy)phenyl)-7,16-dihydro-1,4,5,6,7,8,9,10,13,14,15,16,17,18-tetradecaaza- heptacene in *d*$_1$-TFA with assignments (also based on HMBC and HSQC – see Supplementary Figs. S39 and S40). Carbons shown in orange are provisionally assigned based on proximity of C10 to NH and similarity of environments about C8,C9.

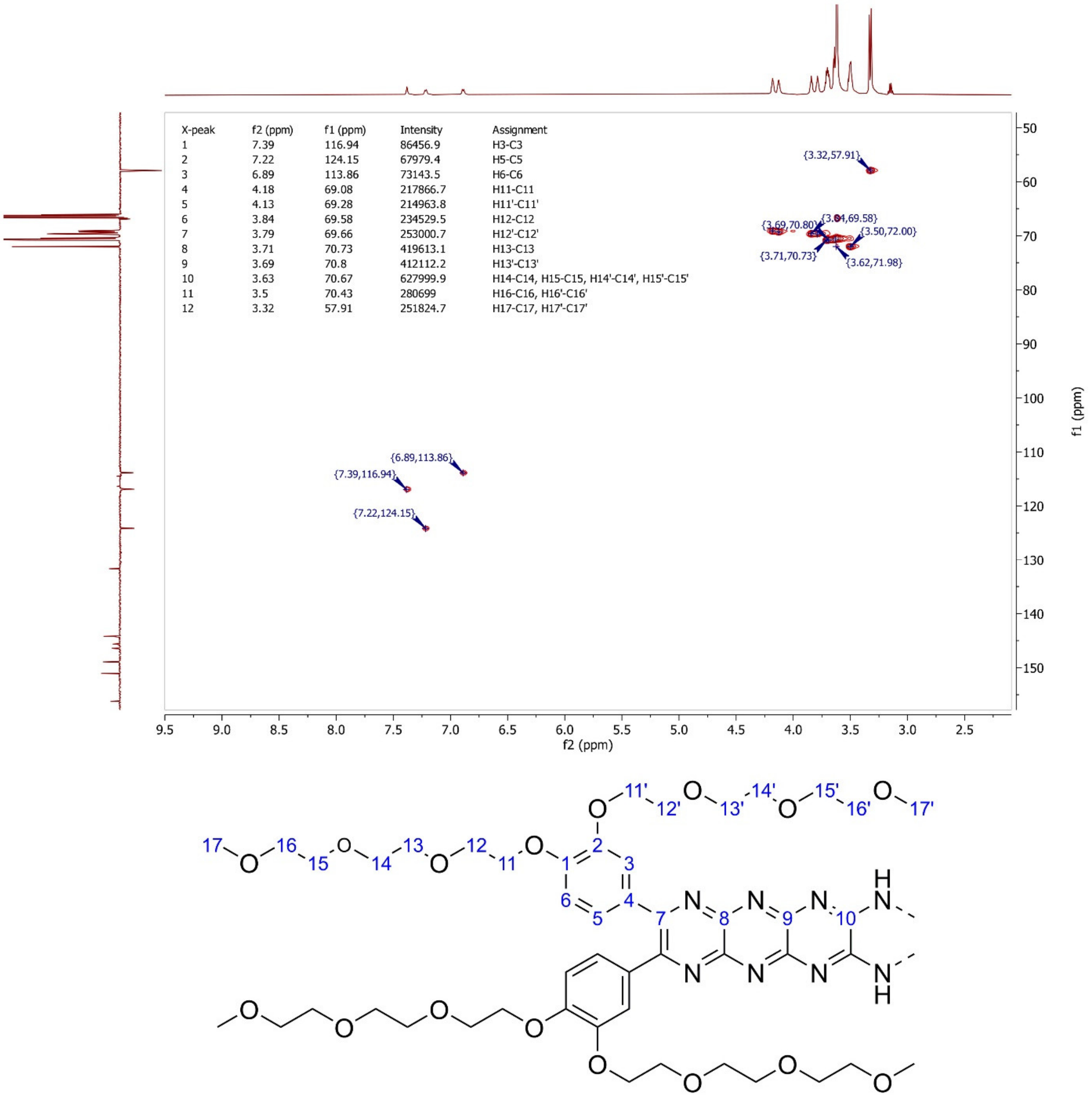

Fig. S38. ^{1}H-^{13}C **heteronuclear single quantum coherence (HSQC) spectrum of 2,3,11,12-tetrakis(3,4-bis(2-(2-(2-methoxyethoxy)ethoxy)ethoxy)phenyl)-7,16-dihydro-1,4,5,6,7,8,9,10,13,14,15,16,17,18-tetra- decaazaheptacene in** d_1**-TFA with assignments.**

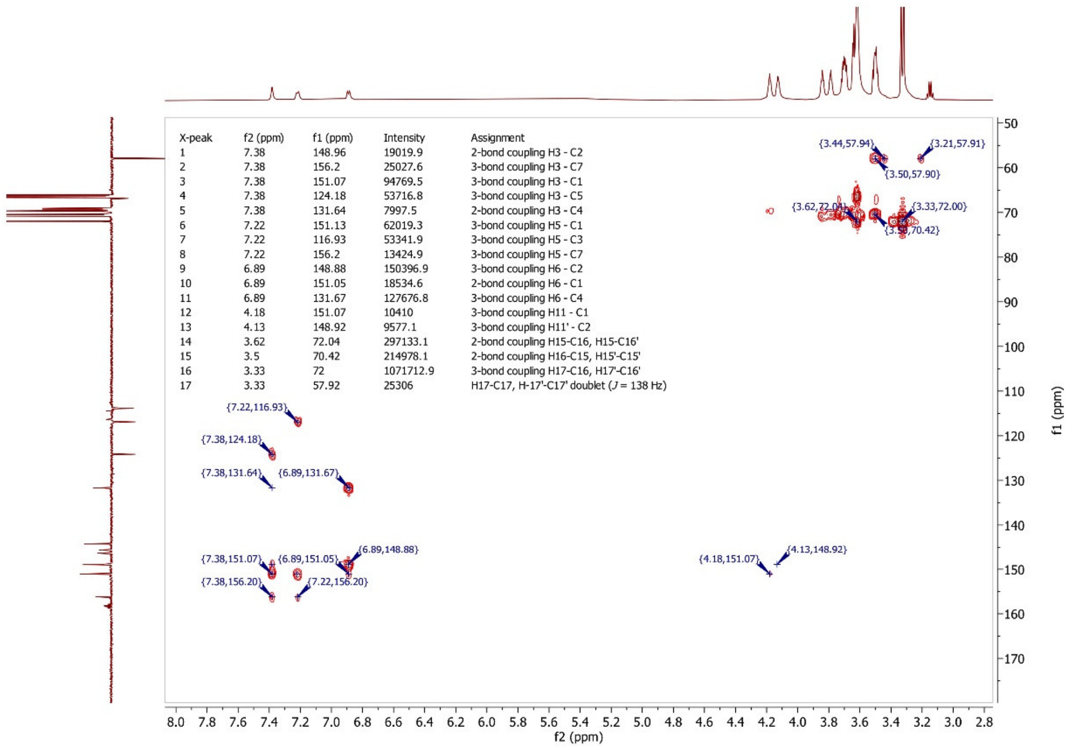

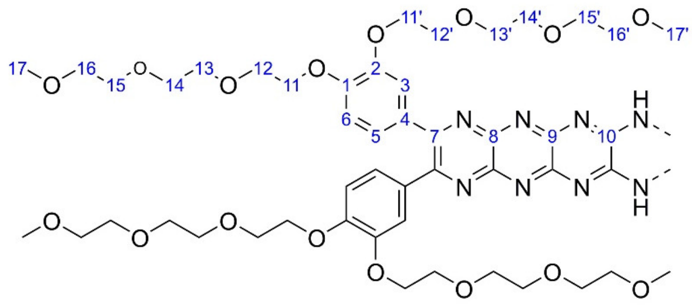

Fig. S39. **^{1}H-[13]C Heteronuclear Multiple Bond Correlation (HMBC) spectrum of 2,3,11,12-tetrakis(3,4-bis(2-(2-(2-methoxyethoxy)ethoxy)ethoxy)phenyl)-7,16-dihydro-1,4,5,6,7,8,9,10,13,14,15,16,17,18-tetra- decaazaheptacene in d_1-TFA with assignments.**

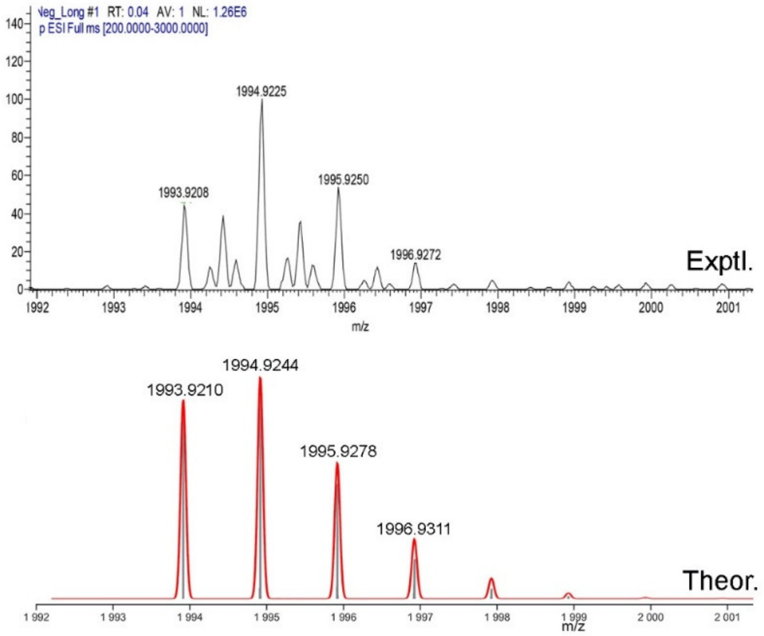

Fig. S40. **Negative ion ESI-TOF-MS spectrum of 2,3,11,12-tetrakis(3,4-bis(2-(2-(2-methoxyethoxy)ethoxy)ethoxy)phenyl)-7,16-dihydro-1,4,5,6,7,8,9,10,13,14,15,16,17,18-tetradecaaza- heptacene.** Upper: experimental spectrum; lower: theoretical spectrum. Theoretical spectrum was generated for $C_{96}H_{133}O_{32}N_{14}$ ($[M - H]^+$) using the tool provided on the Prot-pi website (https://www.protpi.ch).

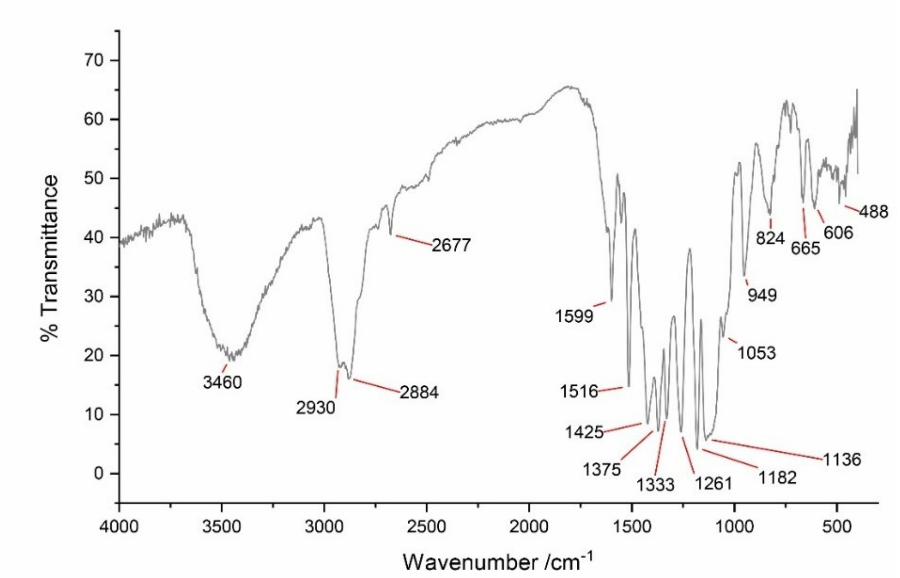

Fig. S41. Fourier transform infrared (FTIR) spectrum (KBr pellet) of 2,3,11,12-tetrakis(3,4-bis(2-(2-(2-methoxyethoxy)ethoxy)ethoxy)phenyl)-7,16-dihydro-1,4,5,6,7,8,9,10,13,14,15,16,17,18-tetradecaaza- heptacene.

**Compound**	**$\lambda_{abs\ max}$ (nm)**	**$\lambda_{em\ max}$ (nm)**	**ε_{max} ($M^{-1}cm^{-1}$)**	**Φ_{PL}**	**Molecular brightness**	**Reference**
TEG_8-N14	664	746	0.3×10^5	$\underline{< 0.01}$	1.5×10^2	This work
TEG_8-N14 in the presence of CTAB	730	750	2.4×10^5	$\underline{0.23}$	5.6×10^4	This work
PC5	630	652	1.5×10^5	0.04	0.6×10^4	*(13)*
Carboxy-SiR	645	667	1.0×10^5	0.40	4.0×10^4	*(16)*
4-carboxyrhodamines (4-642CP)	648	680	1.2×10^5	0.40	4.6×10^4	*(18)*
ATTO647	649	662	1.7×10^5	0.20	3.4×10^4	*Integrated DNA Technologies*
OF650	649	669	1.3×10^5	0.44	5.5×10^4	*(19)*
Cy 5	649	666	2.5×10^5	0.20	5.0×10^4	*Biotechne*
SMSiR	652	670	1.2×10^5	0.31	3.7×10^4	*(17)*
ATTO700	700	716	1.2×10^5	0.25	3.0×10^4	*ATTO-TEC GmbH*
PREX710	712	740	0.9×10^5	0.13	1.2×10^4	*(20)*
trans-1-Halo	715	741	0.9×10^5	0.12	1.0×10^4	*(21)*
SaraFluor 720	721	740	1.6×10^5	0.05	0.8×10^4	*Goryo Chemical*
Alexa Fluor 750	749	775	2.9×10^5	0.12	3.5×10^4	*Thermofisher Scientific*
IR-800CW	774	789	2.4×10^5	0.09	2.2×10^4	*(15)*

Table S1. Comparison of photophysical properties with recently published NIR dye in aqueous solution. Photophysical properties of TEG_8-N14 with or without addition of CTAB were measured in PBS (10 mM, pH 7.4), n=3, two independent experiments. $\lambda_{abs\ max}$, wavelength of maximal absorption; $\lambda_{em\ max}$, wavelength of maximal emission; ε, molar extinction coefficient at $\lambda_{abs\ max}$; Φ_{PL}, fluorescence quantum yield determined using an absolute photoluminescence spectrometer (measured in this study using R6G as a reference during the measurement); molecular brightness is calculated as the molar extinction coefficient multiplied by the quantum yield.

**Compound**	Φ_{PL}
TEG$_8$-N14	0.005 ± 0.001
TEG$_8$-N14/RNA	0.008 ± 0.001
TEG$_8$-N14/dsDNA	0.010 ± 0.001
TEG$_8$-N14/ssDNA	0.011 ± 0.001
TEG$_8$-N14 in the presence of CTAB	0.230 ± 0.003
TEG$_8$-N14/RNA in the presence of CTAB	0.250 ± 0.001
TEG$_8$-N14/dsDNA in the presence of CTAB	0.259 ± 0.001
TEG$_8$-N14/ssDNA in the presence of CTAB	0.264 ± 0.001

Table S2. Fluorescence quantum yield of TEG$_8$-N14 with or without addition of CTAB upon binding to nucleic acid. Photoluminescence quantum yield with excitation of 730 nm was measured in TE buffer in the presence of commercially available calf-thymus dsDNA, calf-thymus ssDNA, and RNA from torula yeast. Φ_{PL}, fluorescence quantum yield determined using an absolute photoluminescence spectrometer. Data presented are mean ± S.D. (n = 3 per experiment).

Movie S1. Confocal images of fixed HeLa cells under a 560 nm excitation wavelength (movie time 00:25).

Movie S2. Confocal images of fixed HeLa cells under a 640 nm excitation wavelength (movie time 00:16).

Movie S3. Confocal images of fixed HeLa cells under a 660 nm excitation wavelength (movie time 00:13).

Movie S4. Confocal images of fixed HeLa cells under a 730 nm excitation wavelength (movie time 00:05).
